# Long term structural and functional neural changes following a single infusion of Ketamine in PTSD

**DOI:** 10.1038/s41386-023-01606-3

**Published:** 2023-06-03

**Authors:** Or Duek, Nachshon Korem, Yutong Li, Ben Kelmendi, Shelley Amen, Charles Gordon, Madison Milne, John H. Krystal, Ifat Levy, Ilan Harpaz-Rotem

**Affiliations:** 1 Department of Psychiatry, Yale University School of Medicine, New-Haven, CT USA; 2 The National Center for PTSD, VA CT Healthcare System, West Haven, CT USA; 3 Departments of Comparative Medicine and Neuroscience, Yale University School of Medicine, New-Haven, CT USA; 4 Department of Psychology, Yale University, New Haven, CT USA; 5 Wu Tsai Institute, Yale University, New Haven, CT USA

**Keywords:** Predictive markers, Consolidation

## Abstract

NMDA receptor antagonists have a vital role in extinction, learning, and reconsolidation processes. During the reconsolidation window, memories are activated into a labile state and can be reconsolidated in an altered form. This concept might have significant clinical implications in treating PTSD. In this pilot study we tested the potential of a single infusion of ketamine, followed by brief exposure therapy, to enhance post-retrieval extinction of PTSD trauma memories. 27 individuals diagnosed with PTSD were randomly assigned to receive either ketamine (0.5 mg/kg 40 min; *N* = 14) or midazolam (0.045 mg/kg; *N* = 13) after retrieval of the traumatic memory. 24 h following infusion, participants received a four-day trauma-focused psychotherapy. Symptoms and brain activity were assessed before treatment, at the end of treatment, and at 30-day follow-up. Amygdala activation to trauma scripts (a major biomarker of fear response) served as the main study outcome. Although PTSD symptoms improved equally in both groups, post-treatment, ketamine recipients showed a lower amygdala (−0.33, sd = 0.13, 95%HDI [−0.56,−0.04]) and hippocampus (−0.3 (sd = 0.19), 95%HDI [−0.65, 0.04]; marginal effect) reactivation to trauma memories, compared to midazolam recipients. Post-retrieval ketamine administration was also associated with decreased connectivity between the amygdala and hippocampus (−0.28, sd = 0.11, 95%HDI [−0.46, −0.11]), with no change in amygdala-vmPFC connectivity. Moreover, reduction in fractional anisotropy in bi-lateral uncinate fasciculus was seen in the Ketamine recipients compared with the midazolam recipients (right: post-treatment: −0.01108, 95% HDI [−0.0184,−0.003]; follow-up: −0.0183, 95% HDI [−0.02719,−0.0107]; left: post-treatment: −0.019, 95% HDI [−0.028,−0.011]; follow-up: −0.017, 95% HDI [−0.026,−0.007]). Taken together it is possible that ketamine may enhance post-retrieval extinction of the original trauma memories in humans. These preliminary findings show promising direction toward the capacity to rewrite human traumatic memories and modulate the fear response for at least 30 days post-extinction. When combined with psychotherapy for PTSD, further investigation of ketamine dose, timing of administration, and frequency of administration, is warranted.

## Introduction

Reactivation of a stored memory (i.e., bringing the previously encoded memory back into consciousness), might change its state from consolidated into a labile, in which its content or meaning may be altered. In a process known as reconsolidation, the memory is then stored again in this altered form [1]. This process is especially relevant to post-traumatic stress disorder (PTSD) as translational theories link the disorder to fear learning and updating [2, 3]. One of the signature symptom clusters in PTSD comprises overgeneralization of fear and occasional re-experiencing of the traumatic memory with its original emotional intensity and vividness. Memory retrieval, either spontaneous or via deliberate exposure [4], may open a time window in which adaptive or maladaptive memory modifications can promote either attenuation or persistence of PTSD symptoms [1, 5].

In the laboratory, fear learning is typically modeled using a paradigm in which neutral cues are paired with aversive outcomes (e.g, electric shocks) and acquire aversive value [6]. Following fear acquisition, repeated presentation of cues in the absence of outcome leads to the extinction of fear responses. But these responses usually return, either spontaneously or following further exposure to the cue or the aversive outcome [7, 8], suggesting that the original memory is not substantially altered. Extinction is likely achieved through inhibition of amygdala fear responses by the ventromedial prefrontal cortex (vmPFC) [9]. However, if extinction training is conducted within the reconsolidation window (at least 10 min but less than 6 h after reactivation [10, 11]), attenuation of fear can be long-lasting [8, 9] and likely involves modification of the original memory [12, 13]. Such post-retrieval extinction does not appear to rely on prefrontal mechanisms [14, 15], and may directly target the memory trace in the amygdala [1, 16, 17]. Similar mechanism may operate in exposure therapy when the trauma memory is recalled into a labile state. During this state the traumatic memory is processed with the therapist so it can be re-consolidated in a new form [18]. This process helps the patient to understand that they are not re-exposed to the *actual* event, they are in a safe environment, and that the original emotional memory-traces in the brain can be altered.

Functional connectivity between the amygdala and hippocampus may also play an important role in the processing of aversive memories [15, 19, 20]. In humans, the functional coupling of the amygdala and hippocampus was associated with the return of fear, with higher coupling after extinction that occurred outside of the reconsolidation window, compared to extinction within the reconsolidation window [15]. In rodents, increased coupling between dorsal hippocampus and amygdala basolateral nuclei was observed during non-REM sleep following a threat task [19].

Anatomical connectivity was also found to play a role in fear conditioning. In vivo magnetic resonance diffusion tensor imaging (DTI) in rodents showed alterations in fractional anisotropy (FA) in the amygdala and hippocampus following fear conditioning. In humans, the uncinate fasciculus (UNC), a white matter bundle connecting the orbito-frontal cortex with limbic structures within the anterior temporal lobe (i.e., amygdala and hippocampus) [21], was found to be related to fear conditioning. Rapid increase in right UNC FA was found to be correlated with decreased response to conditioned stimulus [22]. In contrast, reduced UNC FA was found in people with PTSD diagnosis [23] as well as subthreshold PTSD [24].

Extinction may be further enhanced with the use of ketamine, a non-competitive N-methyl-D-aspartate glutamate receptor (NMDAR) antagonist. The NMDAR has a key role in learning, extinction, and reconsolidation in both animals and humans [25–27]. Moreover, accumulating evidence suggests that on the molecular level, ketamine, in sub-anesthetic doses, promotes neurogenesis [28, 29], cell proliferation [30], and synaptogenesis [29, 31], all of which are important in reconsolidation processes [32, 33]. On the white matter bundle level, ketamine was found to promote a rapid (hours) increase in white matter [34], with a decline over longer periods (months) [35, 36]. However, long-term changes in white matter were tested only with chronic use of ketamine and not with sub-anesthetic dose. A recent study reported a dramatic decline in PTSD symptoms following multiple ketamine infusions, compared to midazolam [37], but the effect was only transient with a median time for loss of response of 27.5 days.

Recent studies have demonstrated the potential of post-retrieval extinction - using behavioral and pharmacological agents - to reduce fear responses. In spider phobia, repeated exposure to a spider image after fear reactivation attenuated amygdala response to that image 24 h later [38]. Administering propranolol (a β-blocker) after reactivation of fear of spiders resulted in decreased avoidance response and increased approach behavior in spider-fearful people [39]. Lastly, administration of propranolol before exposure therapy yielded a steeper decline in PTSD symptoms at follow-up [40]. Taken together, an intriguing possibility is that combining ketamine infusion with psychotherapy will have a synergistic effect [41]. The idea of combining ketamine with psychotherapy was also examined in pain and other mental disorders. Recent systematic review on ketamine-assisted psychotherapy (KAP) found heterogeneity in the use and administration of KAP, with some studies administering ketamine before, during or after the psychotherapy. Results suggest that psychotherapy might prolong the short-term effect of ketamine [42]. In PTSD, a small (*n* = 5 in each group), randomized control trial combining one time ketamine infusion with Trauma Interventions using Mindfulness Based Extinction and Reconsolidation (TIMBER) psychotherapy (3 session in first week, and 9 weekly sessions) found better response and prolonged effect to the TIMBER + ketamine group, compared to control [43].

In this initial investigation, we explored the ability of a single subanesthetic intravenous infusion of ketamine (0.5 mg/kg over 40 min) to enhance post-retrieval extinction of real traumatic memories. To control for the subjective effects of ketamine, we randomized individuals with PTSD to receive an infusion of either ketamine or the benzodiazepine midazolam. We have used active control in order to keep the participants blind. The use of midazolam in such studies and reasoning was reported and discussed in previous published work [37, 44]. As ketamine was found to promote reconsolidation processes, the infusion was followed by an extinction-reconsolidation focused four-day exposure-based therapy (which was initiated 24 h post-infusion while BDNF level peaks [28, 29]).

The aim of this novel pilot exploration was to identify neural biomarkers for post-retrieval extinction of the original traumatic memory for a potential translational clinical trial. Our main hypothesis focused on the amygdala; we predicted a greater reduction in amygdala reactivity to trauma cues in individuals with PTSD who received ketamine, compared to those who were administered midazolam. As the target-engagement mechanism was of reconsolidation-based extinction (and not classic extinction), we expected no change in vmPFC activation or its functional coupling with the amygdala. Moreover, we hypothesized that if ketamine produces greater post-retrieval extinction than midazolam, this effect will also be associated with a reduction in functional connectivity between the amygdala and the hippocampus. We also hypothesized that ketamine will induce alterations on white matter bundles connecting the frontal and temporal lobes. Lastly, we hypothesized that ketamine will be associated with greater reduction in PTSD symptoms than midazolam when combined with exposure treatment.

## Methods and Materials

### Participants

Of the 118 participants who signed informed consent and screened, 33 were eligible and 28 were randomized (see CONSORT in Supplemental). One subject voluntarily left the experiment immediately after the infusion, resulting in 27 participants who were included in the analyses (mean age = 37.8, SD = 10.7, range = 24–63; 14/13 subjects in ketamine/midazolam groups respectively; females (*n* = 10), males (*n* = 17)). All participants had chronic PTSD (more than 1-year), with the average time since trauma being 12.11(∓8.62) years in the ketamine group and 13.88(∓11.18) years in the midazolam group. Out of the 28 participants, two identified as Black, two as Hispanic, one as American Indian, one as Asian and one as Native Hawaiian. Two didn’t state their race or ethnicity. See Table 1 for sample characteristics.

**Table 1:** Descriptive and demographic data.

Variable Mean (∓SD)	Ketamine	Midazolam	Statistics (*df*),*p*
Gender (M/F)	10/4	7/6	Χ^2^*(1)* = 0.16, *p* = n.s
Age	40.7 (∓10.7)	35.1 (∓10.34)	t*(24)* = 1.35, *p* = n.s
Race/Ethnicity	Black - 1	Black - 1	Χ^2^*(5)* = 3.04, *p* = n.s
	Hispanic - 2	Hispanic - 0	
	American Indian - 0	American Indian - 1	
	Asian - 0	Asian - 1	
	Hawaiian - 1	Hawaiian - 0	
	White - 11	White - 10	
	Unknown - 1	Unknown -1	
PCL-5 Before Treatment	48.8 (∓12.3)	44.4 (∓14.4)	t*(25)* = 0.85, *p* = n.s
PCL-5 At End of Treatment	29.5 (∓20.7)	35.1 (∓16.8)	t*(25)* = −0.76, *p* = n.s
PCL-5 Follow-up	32.7 (∓14.95)	28.1 (∓18.1)	t*(21)* = 0.66, *p* = n.s
BDI-II Before Treatment	24.4 (∓9.2)	24.6 (∓12.3)	t(24) = −0.05, *p* = n.s.
BDI-II At End of Treatment	16.5 (∓12.9)	17.1 (∓10.7)	t(25) = −0.12, *p* = n.s.
BDI-II Follow-up	20.9 (∓12.2)	14.5 (∓11.1)	t(22) = 1.4, *p* = n.s.
Time from Criteria A Trauma (years)	12.11 (∓8.62)	13.88 (∓11.18)	t*(23)* = −0.45, *p* = n.s
Lifetime Substance Use/Dependence (yes/no)	7/7	6/7	Χ^2^*(1)* = 1, *p* = n.s
Current MDD Episode	13/13	8/11	Χ^2^*(1)* = 1.94, *p* = n.s
Trauma Type			
Combat	4	0	
Violence	3	2	
Sexual	3	4	
Other	3	5	

The study was registered in clinicaltrials.gov (NCT02727998). Important to notice that while the primary outcome registered in the clinicaltrial.gov was PCL scores, one of the main outcomes in the NARSAD Independent Investigator Award (submitted before trial registration and data collection) was neural biomarker (see Supplemental material Original funded NARSAD grant application Aim 2). The neural mechanize is crucial toward an NIH grant application but this outcome was not registered in error.

Exclusion criteria included a diagnostic history of bipolar disorder, borderline personality disorder, obsessive-compulsive disorder, schizophrenia or schizoaffective disorder, or current psychotic features as determined by the Structured Clinical Interview for DSM-IV (SCID) [45]; dementia was also an exclusion criterion, as were current suicide risk, moderate or higher severity of substance use disorder in the 3 months prior to randomization, and history of mild-to-severe traumatic brain injury (TBI). Participants who were currently engaged in trauma focus therapy were also ineligible to participate in the study. Lastly, patients were excluded for acute medical illness. PTSD diagnosis was established using the Clinician-Administered PTSD Scale (CAPS-5) [46]. The PTSD Checklist for DSM-5 (PCL-5) was used to monitor changes in PTSD symptoms over time [47].

### Randomization

Randomization was managed by the Investigational Drug Services (IDS) Pharmacy at Yale New Haven Health, which also prepared the study drugs for infusion. The entire study team was blind to the conditions. Participants were randomized in counterbalanced blocks of 10-subjects each stratified by gender.

### Retrieve and reprocess exposure therapy

The therapy used in this study consisted of five sessions in total. The first session, conducted before the MRI scans, was a 2 h long meeting where the patient’s life history was taken with a focus on the index trauma. The session also included information on the disorder and the building of an exposure hierarchy, following the guidelines outlined in the prolonged exposure manual [45]. The following four sessions were conducted by an experienced therapist (O.D., I.H.R), each lasting 90–120 min. During the session patients were asked to recall and discuss their traumatic memory through imaginal exposure. The therapy also included processing of the trauma, during which the therapist and the patient discussed the implications of the trauma and ways to reframe the patient’s understanding of the event, and their behavior during the event. Additionally, during each of these four sessions, in-vivo exposure was conducted with a member of the research team, based on the exposure hierarchy constructed with the therapist. The length of psychotherapy was based on two major factors: (1) we wanted to harvest the long-term effects of a single infusion of ketamine, which diminishes after seven days (see discussion) and (2) the promising results of a one-week trauma-focused psychotherapy intervention [48] which allowed us to condense the entire study protocol to accommodate a regular working week.

### Procedure

Eligible participants completed an imagery-development procedure in which they described the traumatic event associated with their PTSD (Criteria A), as well as a sad event and an event in which they felt relaxed for details on the procedure, see [49] for details. Using this information, we developed a 120 s audiotape script of each event, narrated by a male member of the research staff.

**Fig. 1:**
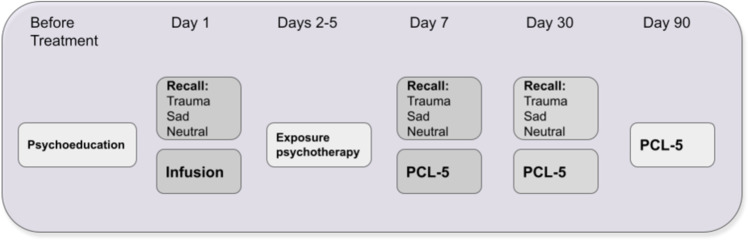
Illustration of the procedure. Days 3–6 = days 3,4,5 and 6 in which the PE sessions were conducted. Recall procedure inside the magnet included 3 rounds of each audio recall script (traumatic, sad, and relax, 120 seconds each).

A day after psychoeducation and building an in-vivo exposure hierarchy session, participants were scanned in the MRI during recall of their traumatic, sad and relax event. The scripts were presented three times, in a fixed order, to avoid ending with the traumatic script. Immediately following script replay, infusion of either ketamine (0.5 mg/kg) or midazolam (0.045 mg/kg) began inside the MRI and lasted for 40 min. The following four days included exposure-based psychotherapy with an experienced clinician (OD, or IHR) in which trauma memory was recalled and reconsolidated after processing. The psychotherapy sessions were accompanied by in-vivo exposure, corresponding to the patients’ avoidance behavior. A week after infusion, participants went through an MRI with a similar procedure to the baseline, excluding the infusion itself. Lastly, we followed participants up for 30 and 90 days. Due to COVID restrictions, 90 days follow-up was mostly done using remote questionnaires, so MRI data is valid for the baseline, 7 and 30 days. See Fig. 1 for study procedure. The study was approved by the Yale University Institutional Review Board (IRB; 1509016530).

### MRI Scans

MRI data were collected with a Siemens 3 T Prisma scanner, using a 32-channel receiver array head coil. High-resolution structural images were acquired by Magnetization-Prepared Rapid Gradient-Echo (MPRAGE) imaging (TR = 1.9 s, TE = 2.77 ms, TI = 900 ms, flip angle = 9°, 176 sagittal slices, voxel size = 1 × 1 × 1 mm, 256 × 256 matrix in a 256 mm FOV). Functional MRI scans were acquired while the participants were listening to the narrated scripts, using a multi-band Echo-planar Imaging (EPI) sequence (multi-band factor = 4, TR = 1000 ms, TE = 30 ms, flip angle = 60°, voxel size = 2 × 2 × 2 mm, 602 mm-thick slices, in-plane resolution = 2 × 2 mm, FOV = 220 mm). For the diffusion-weighted images (DWIs) b-value was set at 1000 s/mm^2^ with an acquisition of a reference image (*b* = 0). 64 directions were scanned, with the phase-encoding gradient applied in the anterior–posterior (AP) direction.

### MRI Preprocessing

Data were preprocessed with Fmriprep, version 1.5.8 [50]. For a complete preprocessing procedure please see supplement 5. For information on motion parameters and the comparison between groups, see supplement 3, Table S1.

### Activation level analysis

For each of the three fMRI sessions, a first-level analysis comparing the first 60 s of the first traumatic script vs. the first 60 s of the first relax script was conducted using FSL 6.0.3 [51], through a Nipype pipeline [52]. We have regressed out DVARS, framewise displacement and the first 6 anatomical component correlations. To avoid capturing habituation effects, we chose to focus on the first 60 s of the first presentation of each script.

The resulting contrasts of parameter estimates (COPE), after z-scoring, were then used to assess specific regions of interest (ROIs) in line with our preliminary hypothesis (i.e., the amygdala, hippocampus, and ventromedial prefrontal cortex (vmPFC)). All ROIs masks were taken from neuroSynth [53]. Mask of each ROI used is presented in the relevant figure.

Each subject’s COPE was masked using the relevant ROI and averaged across all voxels of that region. The extraction of activation for specific ROIs was conducted using Nilearn [54]. Statistical analyses were conducted using python, and Bayesian comparison of groups was done using the pyMC3 package [55]. Lastly, we compared group differences in the same brain regions during the sad (vs. relax) scripts using the same method. This was done to account for an alternative explanation: that the results we see are general to all negative emotions, and not specific to trauma-related ones.

### Functional Connectivity analysis

To assess connectivity between different ROIs, we used the DiFuMO atlas [56], including 256 regions, among them the amygdala, hippocampus (anterior and posterior), and ventromedial prefrontal cortex (vmPFC; and vmPFC anterior; see supplement 1 for region maps). We employed the python package Nilearn [54] to extract time series, using low-pass filtering of 0.1 Hz and high-pass filtering of 0.01 Hz, and regressing out framewise displacement, CSF, white-matter volume, 6 rotation and translations variables, and first six anatomical components based noise correction (CompCor). Then, we extracted the pearson correlation between those regions from the first 60 s of the first traumatic script. We applied pyMC3 [55] to compare changes in correlation between the relevant ROIs (amygdala, anterior and posterior hippocampus, anterior vmPFC, and vmPFC). Lastly, we conducted the same procedure on the first 60 s of the sad script (as we did in the activation analysis), in order to test whether these effects are related to negative emotions in general or to trauma-related ones specifically.

### Diffusion-weighted images analysis

Diffusion-weighted images analysis was done using standard TRACULA (TRActs Constrained by UnderLying Anatomy) protocol [57, 58]. TRACULA is an automatic WM pathways reconstruction tool. It uses global probabilistic tractography [59] with anatomical priors to reconstruct major WM tracts such as the UNC [57]. TRACULA preprocessing includes image corrections for eddy currents, simple head motions, B0 distortion correction, and computation of the diffusion tensor. The affine intra-subject registration with the T1-weighted images was done using Freesurfer’s “bbregister” [60]. Inter-subject registration was done using the affine inter-subject registration to MNI152 space. The TRACULA standard procedures were then performed to fit the ball-and-stick model of diffusion to the diffusion-weighted images and for probabilistic tracking and track segmentation. For each participant, following visual inspection of reconstructed tracts, we extracted diffusion tensor mean fractional anisotropy (FA), estimated with dtifit, in bi lateral uncinate fasciculus. Motion parameter was calculated as the sum of deviations from average rotation and average translation [58] (for information on motion parameters and comparison between groups, see supplement 3, Table S1). Percent bad slices was equal to 0 and average dropout score was equal to 1 in all scans.

### Statistical approach

In accordance with recent guidelines and findings [61–63], analysis in this manuscript was based on a Bayesian approach. DTI analysis was conducted using brms, all other analyses were conducted using PyMC3, probabilistic programming packages in R and python [55], respectively. This also promotes better measures of uncertainty in the findings, which is most critical in small sample sizes [64]. To assess the difference in reactivation (as well as functional connectivity) to the traumatic memory, we have used a Bayesian multilevel model. The model included the ROI (amygdala, hippocampus, vmPFC) activation as the dependent variable. Activation of the ROI at baseline was entered as a covariate into the model (to account for possible random differences between the groups at baseline). The model included the group (ketamine/midazolam) and the interaction of time and group; lastly, subjects were entered as random intercept (to account for the within-subject random effects). As activation at baseline was entered as covariate, we expected to see a group effect (i.e., a difference between ketamine and midazolam at the end of treatment and at follow-up). Here we report the difference between the posterior distribution of each group at T2 (end of treatment) and T3 (30 days).

Effect sizes were calculated by dividing this difference between distributions with the total unexplained variance of the model. A difference was considered robust (i.e., significant) when the 95% High Density Interval (HDI) was excluding zero (i.e. either the interval is completely below or above zero) [65]. To promote better inference, we also report the Bayes-Factor of each of the effect sizes in each analysis. Bayes Factor tests what is the probability that our hypothesis (i.e., the difference between the groups) is true, given the data, compared to the null hypothesis (Bayes Factor 1 over 0, i.e., BF_10_). The calculation gives the reader another aspect of the strength of the evidence. BF_10_ > 3 is considered robust, whereas BF_10_ < 1 suggests that the null hypothesis is more probable.

Assessment the changes in FA in the right and left UNC were done in R, using Bayesian multilevel regression models (BMLMs) as implemented in the ‘brms’ package [66]. HDI were calculated using the coda package [67]. Results are considered robust (i.e., significant) if HDI does not include zero. FA residuals controlling for age, gender, and motion were calculated and baseline corrected to account for initial differences in FA values. In an attempt to improve convergence while reducing overfit, specified mildly informative conservative priors were applied with priors set as a student T distribution mean of 0 and standard deviation of 1 to reduce the effects of outliers [68, 69]. All parameters indicated a good fit of the data (No divergences, Rhat < 1.01 and ESS > 1000).

Originally, based on classic power calculations (using G*power) [70] the required sample size to detect an effect size of 0.8 (commonly observed in psychotherapy) was determined to be 40 (20 for each group), with 90% power. Due to COVID restrictions, data collection was halted earlier. Using the same analysis (G*power), power was reduced to 80%. As we used a Bayesian model, we calculated the relevant power for our fMRI using simulated data. We have simulated a different number of observations per group to test our power (i.e., the proportions of a robust effect, given the sample size). Results suggest that in a sample size of 25, given an effect size of 0.6 (one-tailed hypothesis), our power is 90%. For details on the simulation analysis, see supplement 4. Full analysis scripts can be found here: https://github.com/orduek/KPE.

## Results

### Changes in PTSD symptoms

PCL-5 scores were recorded before treatment, at the end of treatment, and at 30 and 90 days follow-up. Although PTSD symptoms significantly and robustly improved over time [before treatment, *M* = 46.6, sd = 13.35 end of treatment (7 days after infusion): *M* = 33.8, sd = 18.37; 30-day follow-up: *M* = 30.79, sd = 15.79, 90-day: *M* = 31.04, sd = 17.55] there was no significant difference between the ketamine and midazolam groups in the rate of improvement or in the PTSD score at the end of treatment and follow-up (mean difference = 0.41, 95% HDI [−1.42, 2.31]). Comparing symptoms at the end of treatment with symptoms before treatment showed significant difference (mean difference = 12.56, 95%HDI [21.84,3.15], effect-size [95%HDI] = 0.8 [0.23,1.39], BF_10_ = 10.65). Comparing 30 days follow-up to baseline showed significant difference as well (mean difference = 16.1, 95%HDI [25.33, 6.48], effect-size [95%HDI] = 1.00[0.40,1.60] BF_10_ = 35.76). The 90 days follow-up showed stability with difference from before treatment = 15.9 95%HDI [25.74,6.35], effect-size [95%HDI] = 0.98[0.38,1.60], BF_10_ = 32.59; supplement 2, fig. S1.

### Neural m echanism

Based on our hypotheses, the main analysis focused on activation and connectivity patterns of the amygdala. In each ROI (i.e amygdala, hippocampus, vmPFC), we contrasted activation during the traumatic script with activation during the relax script at each timepoint. Here we report the effects derived from the interaction between time and medication group.

### Amygdala reactivity

**Fig. 2:**
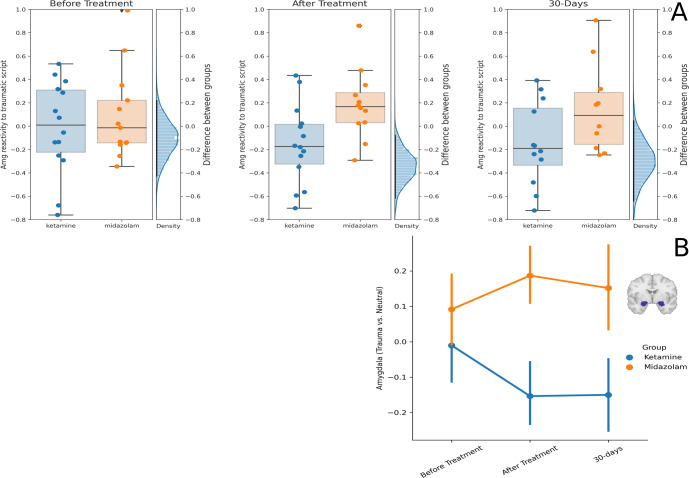
Amygdala Reactivity. **A** Differences between the ketamine and midazolam groups in amygdala reactivity to trauma vs. relax scripts across the three-time points. Each dot is a participant, the horizontal line in the middle of the boxplot represents median. On the right of each panel is the posterior distribution of the difference between the groups. The black line is the 95% HDI. **B** Average amygdala reactivity to trauma vs. relax in the ketamine group (blue) and the midazolam group (orange) in the three-time points. Error bars represent standard error of the mean. Brain image depicts the mask of the amygdala ROI (center: MNI [−22,0,−20], [22,0,−20]).

Activation difference for traumatic vs. relax memories was lower in the ketamine group compared to the midazolam group at the end of treatment, with mean activation of ketamine group −0.15 (sd = 0.33; *N* = 14), mean activation of midazolam group 0.18 (sd = 0.3; *N* = 12) and mean posterior difference: −0.33, sd = 0.13, 95%HDI [−0.56,−0.04]; effect size [95%HDI] = 1.00 [0.16,1.86], BF_10_ = 7.45). This difference was marginally significant at the 30-day follow-up (ketamine: −0.14 (sd = 0.35; *N* = 12), midazolam: 0.15 (sd = 0.38; *N* = 10), mean difference: −0.28, sd = 0.14, 95%HDI [−0.56, 0.01], effect size [95%HDI] = 0.87 [−0.01,1.75], BF_10_ = 2.58; Fig. 2). There were no group differences in amygdala activation during the sad imagery scripts at any time point (supplement 2, fig. S2).

### Hippocampus reactivity

**Fig. 3:**
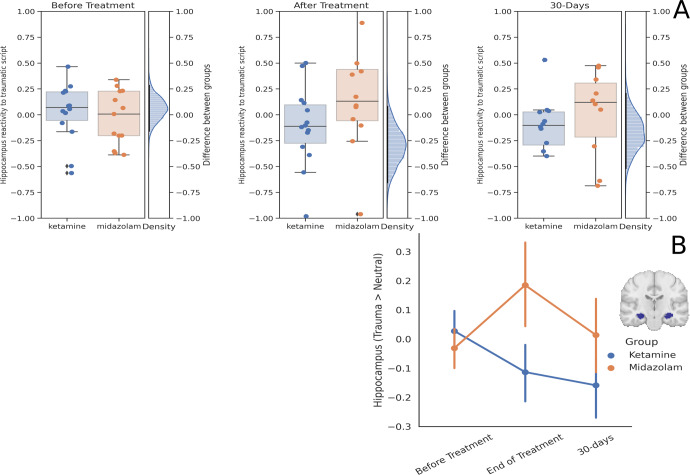
Hippocampus Reactivity. **A** Differences between the ketamine and midazolam groups in hippocampus reactivity to trauma vs. relax scripts across the three time points. Each dot is a participant, the horizontal line in the middle of the boxplot represents median. On the right of each panel is the posterior distribution of the difference between the groups. The black line is the 95% HDI. **B** Average hippocampus reactivity to trauma vs. relax in the ketamine group (blue) and the midazolam group (orange) in the three time points. Error bars represent standard error of the mean. Brain image depicts the mask of the hippocampus ROI (center: MNI [−28,−18,−16], [28,−18,−16]).

Hippocampal activation to trauma vs. relax memories was marginally lower in the ketamine (mean = −0.11, sd = 0.38; *N* = 14) compared to the midazolam (mean = 0.18, sd = 0.53; *N* = 12) group at the end of treatment, with mean difference −0.3 (sd = 0.199), 95%HDI [−0.65, 0.04], effect size [95%HDI] was 0.71 [−0.12, 1.49], BF_10_ = 1.77. There was no group difference in the 30-day follow-up (ketamine: −0.15 (sd = 0.38; *N* = 12), midazolam: 0.01 (sd = 0.42; *N* = 10), mean difference −0.17, sd=0.18, 95%HDI [−0.54, 0.21]), effect size [95%HDI] was 0.39 [−0.48, 1.25], BF_10_ = 0.65; see Fig. 3. There were no group differences in hippocampus activation of the sad imagery script at any time point (supplement 2 fig. S3).

### vmPFC reactivity

We found no group differences in vmPFC activation to traumatic imagery scripts (compared to relax) at any time point. There was no group difference in vmPFC activation during the sad imagery scripts compared to the relax ones at any of the three-time points. For a full description of the vmPFC activation results see supplement 2 fig. S4, S5.

### Amygdala-vmPFC functional connectivity

Functional connectivity (FC) between the amygdala and vmPFC was not significantly different between the groups at any time point. For details, please see supplement 2 fig. S6.

### Amygdala - Hippocampus functional connectivity

Functional connectivity (FC) between the amygdala and posterior hippocampus at the end of treatment was significantly lower in the ketamine group (mean 0.15, sd = 0.32; *N* = 14) than in the midazolam group (mean 0.43, sd = 0.18; *N* = 12), with mean difference: −0.285, sd = 0.11, 95%HDI [−0.50, −0.05], effect size [95%HDI] = 1.06 [0.18,1.92], BF_10_ = 7.59. At 30-days follow-up we found no difference between the ketamine (mean FC: 0.37, sd = 0.26; *N* = 12) and midazolam (mean FC: 0.29, sd = 0.35; *N* = 10) groups (mean difference: 0.08, sd = 0.15, 95%HDI [−0.17, 0.32], effect size [95%HDI] = 0.43 [−1.14.,0.62], BF_10_ = 0.55). A similar analysis of the connectivity between the amygdala and anterior hippocampus revealed no group differences (supplement 2). There were also no group differences in amygdala-posterior hippocampus functional connectivity during the sad imagery script at any of the three-time points (supplement 2 fig. S7).

### Alterations in White matter

**Fig. 4:**
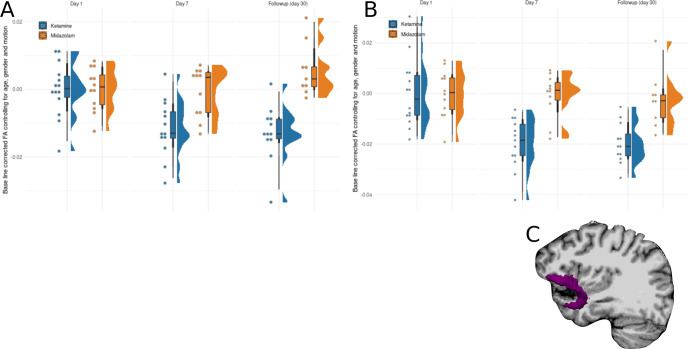
Diffusion Tensor Imaging analysis (DTI). Differences between the ketamine and midazolam groups in the (**A**) right and (**B**) left UNC FA adjusted for age, gender, motion and baseline corrected. Each dot is a participant, boxplots include the median, two hinges and two whiskers, and the density distribution of participants. **C** An illustration of the UNC.

A robust interaction between session (baseline, post-treatment, 30-day follow-up) and drug on FA in the right UNC (post-treatment interaction coefficient: −0.01, 95% HDI [−0.02, −0.002]; follow-up interaction coefficient: −0.01, 95% HDI [−0.013,−0.003]), suggests a differential response to drug across sessions. In order to investigate the interaction, each session was analyzed separately. Ketamine showed a robust reduction in FA in the right UNC compared with midazolam group that lasts for at least 30 days (post-treatment: −0.011, 95% HDI [−0.018,−0.003]; follow-up: −0.018, 95% HDI [−0.027,−0.01]). Figure 4a shows the right UNC WMI corrected for age, gender and motion across time. Analyzing the left UNC, yielded similar results, with a robust interaction between session and drug on FA in the left UNC (post-treatment interaction coefficient: −0.019, 95% HDI [−0.035, −0.004]; follow-up interaction coefficient: −0.017, 95% HDI [−0.032,−0.002]). In order to investigate the interaction, each session was analyzed separately. Ketamine showed a robust reduction in FA in the right UNC compared with midazolam group that lasts for at least 30 days (post-treatment: −0.019, 95% HDI [−0.028,−0.011], BF_10_ = 9.45; follow-up: −0.017, 95% HDI [−0.026,−0.009], BF_10_ = 137). Figure 4b shows the left UNC WMI corrected for age, gender and motion across time.

## Discussion

In this work, we provided preliminary evidence that a single infusion of 0.5 mg/kg ketamine for 40 min, when combined with exposure and processing of the traumatic memory, lead to alterations in neural circuit functioning, although it did not produce reduction in PTSD symptoms greater than midazolam. These changes were more consistent with modification of the original memory (i.e., post-retrieval extinction) than with enhancement of classic extinction. The ketamine group showed lower amygdala reactivity to recalled subjective traumatic events compared to the midazolam group, with no change in connectivity between the amygdala and vmPFC. Moreover, connectivity between the amygdala and posterior-hippocampus was reduced in the ketamine, but not the midazolam group, at the end of treatment. Last, ketamine, but not midazolam, was also associated with long-term reduction in FA in the UNC. The combined results suggest a potential, larger reduction in the level of neuronal reactivity associated with the original trauma memory in the ketamine group compared to midazolam.

The amygdala is a key node in neural networks engaged in extinction and post-retrieval extinction processes [9, 15, 71]. Some studies argue that diminished amygdala activation that is independent of vmPFC inhibitory function, is associated with post-retrieval extinction, which is in line with the classic reconsolidation findings, blocking protein synthesis in the basolateral amygdala (BLA) [1]. Other studies have found that extinction learning involves an inhibitory signal from the prefrontal cortex, mainly the vmPFC, to the amygdala and hippocampus [9]. Taken together, these findings suggest that extinction learning should present with higher connectivity between vmPFC and amygdala and higher activation of the vmPFC. Reconsolidation-based extinction (i.e. post-retrieval extinction), on the other hand, should present with lower activation in the amygdala during retrieval, regardless of vmPFC-amygdala connectivity. Our findings of diminished activation of the amygdala and lack of enhanced connectivity between vmPFC and amygdala, in response to trauma recall in the ketamine group, are consistent with the post-retrieval extinction theory [14]. Interestingly, a recent study reported an association between decreased PTSD symptoms and increased amygdala-vmPFC connectivity during emotional face viewing, especially in participants who received multiple infusions of ketamine [72]. Our results suggest a possible path of combining ketamine with psychotherapy and assessing their effect on the neural mechanisms underlying real autobiographical memories. While ketamine may have a general effect on regulating emotions, our theory proposes that combining it with the retrieval of the traumatic memory may lead to modification of the original traumatic memory through a process called post-retrieval extinction. However, further research is needed to confirm this theory.

NMDARs are required for the transition of memory from a fixed to a labile state. For example, an NMDAR antagonist, but not an AMPAR antagonist, blocked the reconsolidation process [27]. NMDAR activation may be upstream of the stimulation of protein synthesis in the mechanistic cascade responsible for making memories labile, as the addition of anisomycin does not augment the interference with reconsolidation produced by an NMDAR antagonist [1, 27]. Midazolam, a benzodiazepine positive allosteric modulator of GABA_A_ receptors, has also been reported to interfere with the reconsolidation of fear [73, 74]. While some studies have found that benzodiazepine impaired extinction learning [75, 76], others have shown that if benzodiazepine is administered after memory reactivation (i.e., within the reconsolidation window), it blocks the reconsolidation of fear and as such promotes extinction of fear response [73, 74]. As midazolam’s half-life is relatively short (2.3 h) [77], it is highly unlikely that it will interfere with post-retrieval extinction which occurred 24 h post-infusion. Based on the current study procedures, we cannot rule out that some reconsolidation blockade might have occurred in both agents as the infusion was conducted following baseline memory activation task in the MRI. It is thus possible that the effect shown at the end of treatment derives from both the elevation of activation in the midazolam group and the reduced activation in the ketamine group.

Studies in animals show that 90 min following acute subanesthetic dose, ketamine causes reduction in synaptic density (PSD-95) due to the blockade of NMDA receptors [78]. However, 4 h post-injection there is a significant increase in WMI [34]. By 24 h following acute subanesthetic dose, evidence show ketamine promotes neuroplasticity by enhancing glutamate release, raising BDNF levels, activating mTORC1 signaling, producing cortical synaptogenesis, and stimulating hippocampal neurogenesis [28, 29], all of which are important to the reconsolidation processes [32, 33]. Supporting these findings, ketamine-related increase in hippocampal BDNF gene expression reduces reactivation of fear memories in rats [79]. However, the well-known antidepressant [80, 81] and anxiolytic [82] effects of ketamine emerge only 24 h to several days after ketamine administration. This enhanced neural plasticity and synaptic reorganization may be the main mechanism of action underlying our results, as traumatic memories were retrieved during the plastic stage, 24 h post-infusion, and reprocessed in a safe environment. This procedure was repeated for a total of 4 post-infusion daily exposures to further enhance the reconsolidation of the altered memory trace, as BDNF levels only return to baseline after 7 days [83]. In contrast, midazolam’s effect on memory reconsolidation (via GABA-A receptor signaling) appears to last only up to 60 min [84] post-administration.

It was suggested that ketamine does not simply increase connectivity, rather, it promotes the reorganization of the connections [85, 86]. Phoumthipphavong et al. (2016) checking for the effect of a single sub-anesthetic dose on mice dendritic architecture, found increase in synaptic density as well as retraction of distal spines. Reduction in FA in the UNC, represents a similar process i.e., reduced white matter integrity in long range axon bundles. Studies suggest that memories are stored in neuronal ensembles [87, 88]. These ensembles trigger the emotional response. Repeated activation of the memory in a safe environment, where the response is not initiated, alters the behavioral response and thus the ensemble that triggers the memory. As the response, the long range connections over the bundle (UNC) diminishes. It is possible that the interaction of psychotherapy and ketamine yields this reorganization of synapses, but our current design does not allow us to conclude such a thing yet. Further exploration of dosage, frequency and timing of ketamine when combined with psychotherapy is needed.

Our results also show reduced amygdala-hippocampus connectivity following ketamine infusion. This reduction was only apparent 7 days after infusion and not in the follow-up session (30 days), suggesting a transient effect. This finding is consistent with prior research demonstrating a role for amygdala-hippocampus interaction in the acquisition and modification of fear memories [15]. For example, the strength of amygdala-hippocampus connectivity is associated with the ability to encode long-term memories [20, 89–93]. Coupling between the amygdala and dorsal hippocampus during non-REM sleep increases following threat conditioning in rodents [19]. The role of the hippocampus in contextual fear memory was recently highlighted in a study that disrupted the reconsolidation of fear by inhibiting protein synthesis in hippocampal ensemble [94].

In healthy individuals, higher amygdala-hippocampus connectivity may be associated with enhanced memory under stress, but not neutral, conditions [92]. Although initial evidence suggested that anterior (rather than posterior) hippocampus was associated with emotional memories [95], posterior hippocampal connectivity was perturbed in PTSD (compared to healthy controls and Anxiety) in both resting-state and task-based (emotional faces) measures [96]. Moreover, individuals with PTSD had a smaller posterior hippocampus [97], and impaired posterior-anterior hippocampal connectivity [98]. The decreased connectivity between posterior-hippocampus and amygdala found in this investigation may serve as a potential biomarker of reduced fear memory trace following an NMDA enhanced post-retrieval extinction. Results may also point to the role of the posterior-hippocampus in real (context-dependent) traumatic memories.

The facilitation of post-retrieval extinction of trauma memories in this study raises the possibility that behavioral interventions may enhance the reported efficacy of ketamine in the treatment of PTSD [44]. PTSD is a highly disabling disorder with few effective pharmacotherapy options [99]. It is speculated that PTSD derives from overgeneralization of fear responses and excessive or maladaptive reconsolidation of the traumatic memories. As such, reconsolidation-based treatments can be clinically relevant for treating such a disorder. Although some evidence-based treatments for PTSD are highly effective, they reach 50% remission at best [100]. Using ketamine as a facilitator is consistent with a recent study conducted in participants with harmful drinking patterns [101]. While ketamine alone was ineffective in reducing drinking, when administered in conjunction with alcohol cues, ketamine interfered with the reconsolidation of alcohol-reward-related memories and significantly reduced drinking.

Although neural activity indicated a greater reduction in levels of distress associated with the traumatic memory in the ketamine group, we did not find any differences in PTSD symptoms between the experimental groups. A high level of heterogeneity characterizes PTSD symptomatology and thus presents a challenge for detecting nuances in treatment response, more so, in small samples. In this context, other studies which tested the effectiveness of exposure therapy on PTSD or fear of flying reported similar outcome differences in physiological fear response between the experimental groups at the end of the intervention with no difference in the self-report measure of symptoms [102, 103]. It is possible that a subjective account of psychological distress, measured by verbal account of psychiatric symptoms, is less sensitive to change than objective neurobiological measures. This hypothesis is in line with the NIMH Research Domain Criteria (RDoC) initiative to further establish biomarkers associated with mental disorders.

This study has some limitations. First, the number of participants in each group is relatively small, and larger studies will need to replicate the findings. This concern is partially mitigated by the longitudinal aspect of the study, as well as the findings, which are consistent with other animal and human studies. Another potential limitation is the use of an active psychotropic drug, midazolam. We used midazolam as a comparator to control for the subjective effects of ketamine and to preserve the blind conditions. This limits our inferences, however, to the relative effects of similarly tolerable doses of ketamine and midazolam. In order to fully unfold the effect of ketamine, a control group receiving ketamine without recall of trauma is needed. Lastly, it is important to note that since all participants received psychotherapy, it is difficult to determine the specific effect of ketamine alone and the effect of the combination of ketamine and psychotherapy.

While preliminary, the present pilot study finds that one-time ketamine infusion might enhance post-retrieval extinction of traumatic memories. This is apparent in the effects found in the ketamine vs. the midazolam group: diminished amygdala reactivity to recall of traumatic events, reduced WMI in the UNC, and attenuation of connectivity between amygdala and hippocampus. These findings complement previous findings in both animal models and humans, on a wider spectrum of psychiatric disorders and both appetitive and aversive memories. As the enhancement of post-retrieval extinction presented here was demonstrated using real-life traumatic events, the applicability of this procedure is high and it might serve as a potential novel future intervention for PTSD and anxiety disorders.

### Supplementary information

CONSORT

Supplemental

## Data Availability

Data and analysis can be found in the Github repository.

## Code availability

All analysis scripts can be found here https://github.com/orduek/KPE.
