# Supplementary figures and images for: Long term structural and functional neural changes following a single infusion of Ketamine in PTSD
Source: Neuropsychopharmacology. 2023 Jun 3;48(11):1648–58. doi: 10.1038/s41386-023-01606-3 (PMC10517133; doi:10.1038/s41386-023-01606-3)

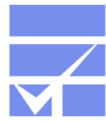

# CONSORT

TRANSPARENT REPORTING of TRIALS

## CONSORT 2010 Flow Diagram

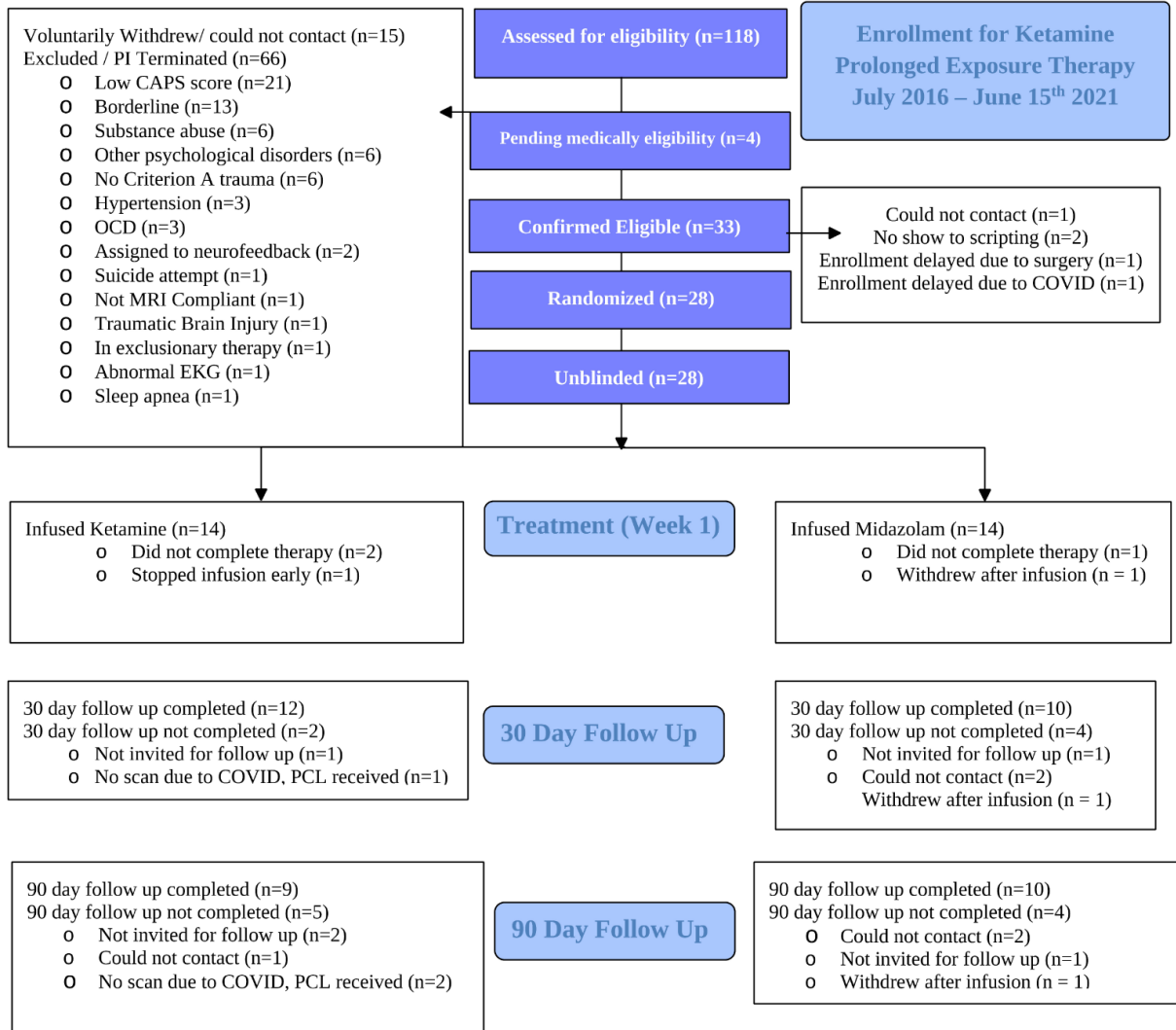

Supplement: Supplementary file 1 — CONSORT [file 41386_2023_1606_MOESM1_ESM.pdf]
