# Supplementary material for: Long term structural and functional neural changes following a single infusion of Ketamine in PTSD
Source: Neuropsychopharmacology. 2023 Jun 3;48(11):1648–58. doi: 10.1038/s41386-023-01606-3 (PMC10517133; doi:10.1038/s41386-023-01606-3)
Supplement: Supplementary file 2 — Supplemental [file 41386_2023_1606_MOESM2_ESM.pdf]

Supplemental Materials:

Supplement 1: DiFuMo regions extracted

Amygdala:

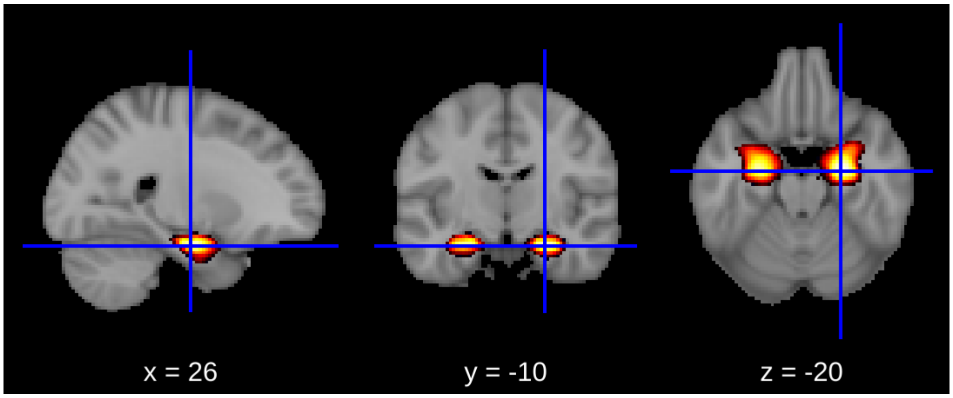

Anterior hippocampus:

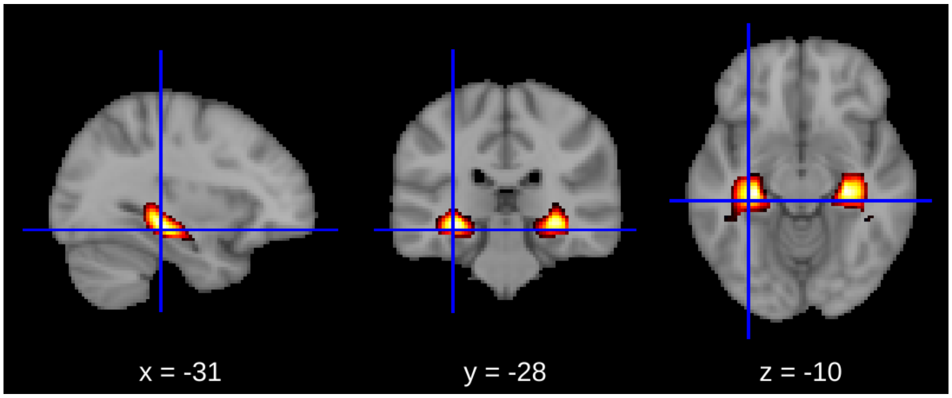

Posterior hippocampus:

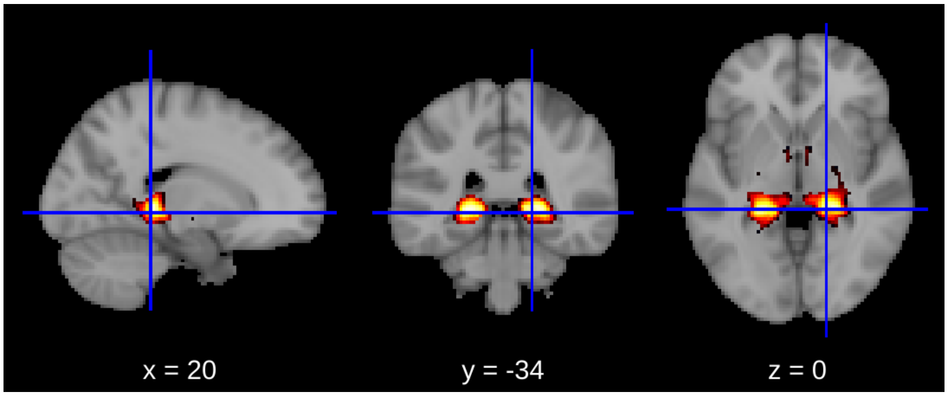

vmPFC:

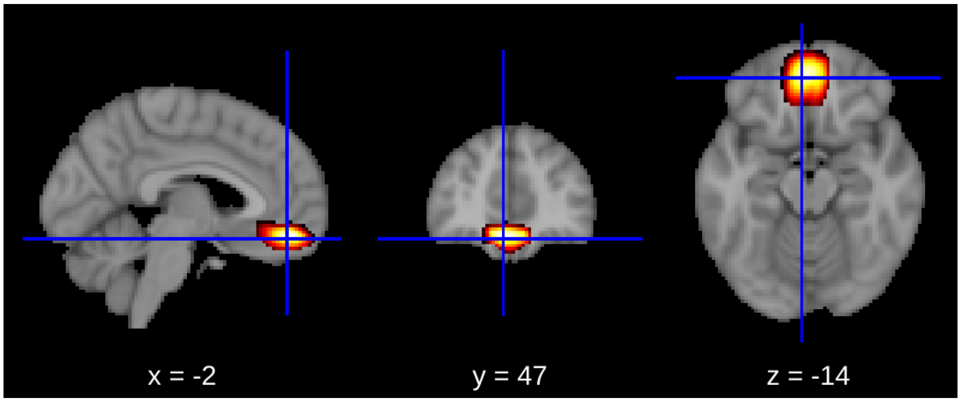

Supplement 2:

Symptoms change

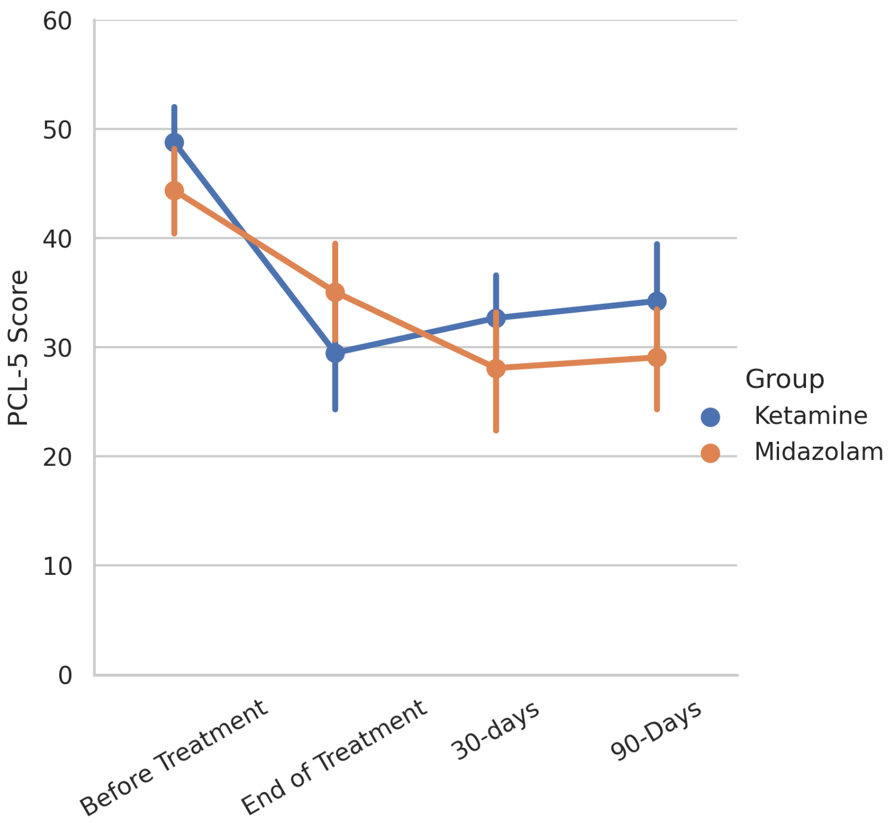

Figure S1: Total PCL-5 scores at each time point in the ketamine (blue) and midazolam (orange) groups. Error bars represent the standard error of the mean.

Sad vs. Neutral contrast results: Changes in neural activation

Amygdala:

Comparing the amygdala activation while listening to the sad script (vs. neutral) revealed no groups differences after treatment [ketamine M=0.10 (sd=0.28], midaolzam: 0.08 (sd=0.35), mean posterior=0.007 (sd=0.12) 95%HDI (-0.22,0.24)]; and in 30-day follow-up [ketamine M=0.01 (sd=0.24], midaolzam: -0.04 (sd=0.28), mean posterior = 0.055 (sd=0.12) 95%HDI (-0.19,0.31)]. Although no difference was found between the groups, there was an effect for time. Comparing the amygdala activation of both groups in the 30-day followup to the one before treatment revealed a marginal decline in activation (overlapping of posterior distributions was 0.052). There was no significant decline 7-days after infusion (see figure below).

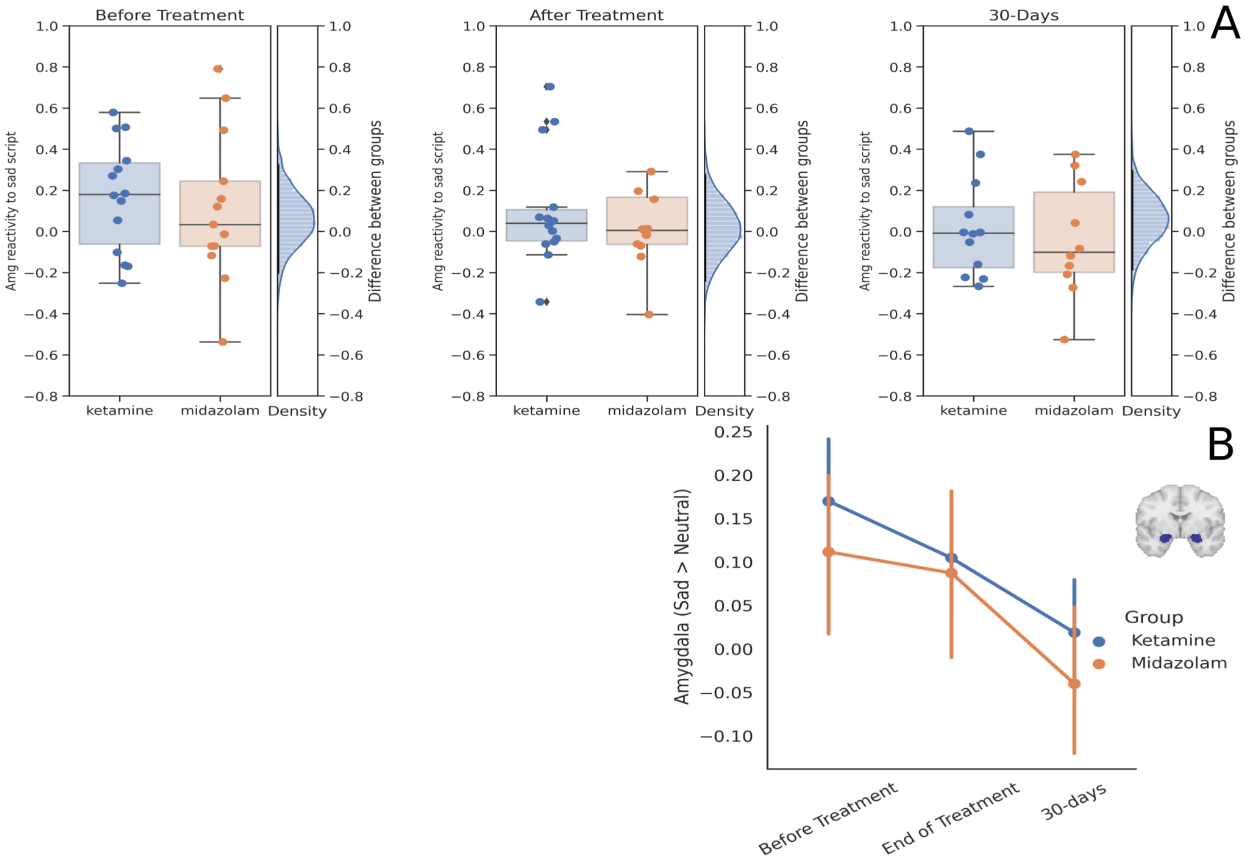

Figure S2: (a) Differences between the ketamine and midazolam groups in amygdala reactivity to sad vs. relax scripts across the three-time points. Each dot is a participant, the horizontal line in the middle of the boxplot represents median. On the right of each panel is the posterior distribution of the difference between the groups. The black line is the 95% HDI. **(b)** Average amygdala reactivity to sad vs. relax in the ketamine group (blue) and the midazolam group (orange) in the three-time points. Error bars represent standard error of the mean. Brain image depicts the mask of the amygdala ROI (center: MNI [-22,0,-20], [22,0,-20])

Hippocampus:

Comparing the hippocampus activation while listening to the sad script (vs. neutral) revealed no groups differences after treatment [ketamine M=0.11 (sd=0.27], midaolzam: 0.01 (sd=0.37), mean posterior=0.103 (sd=0.13) 95%HDI (-0.15, 0.34)]; and in 30-day follow-up [ketamine M=-0.07 (sd=0.32], midaolzam: -0.05 (sd=0.24), mean posterior = -0.02 (sd=0.13) 95%HDI (-0.29, 0.28)]. Although no difference was found between the groups, there was an effect for time. Comparing the hippocampus activation of both groups in the 30-day followup to the one before treatment revealed a significant decline in activation (overlapping of posterior distributions was 0.01). There was no significant decline 7-days after infusion (see figure below).

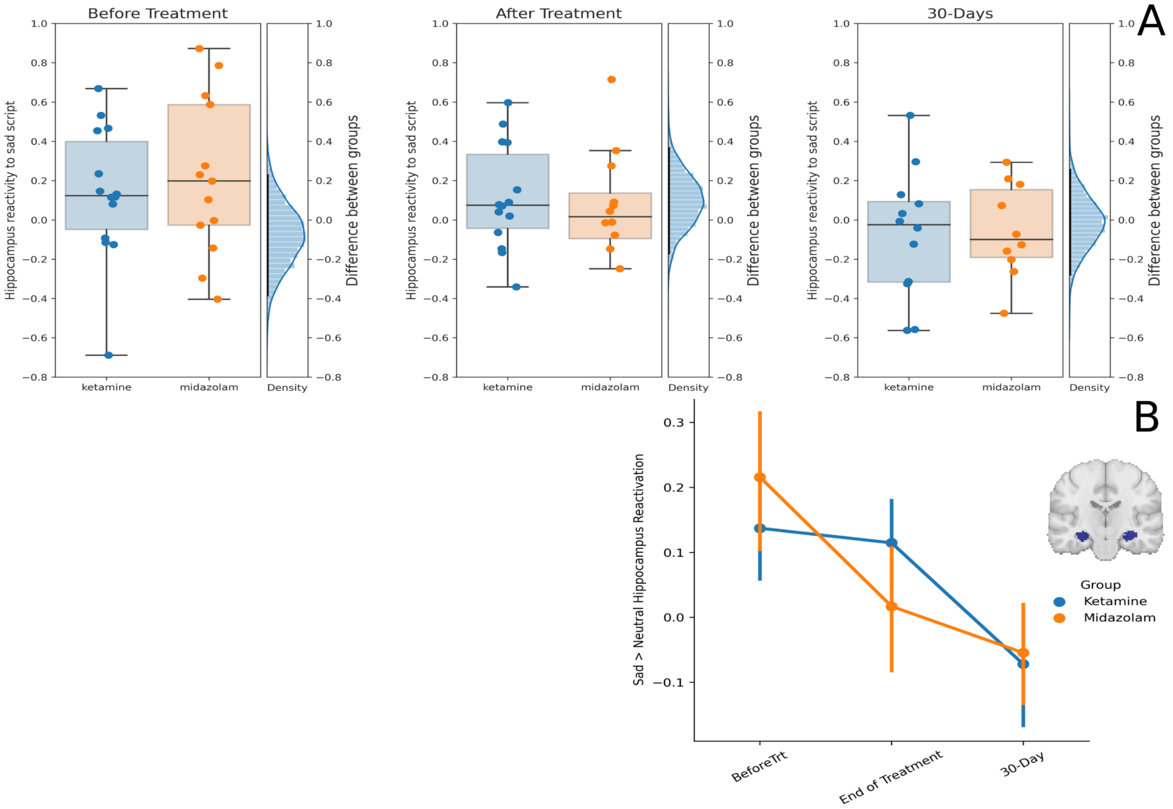

Figure S3: (a) Differences between the ketamine and midazolam groups in hippocampus reactivity to sad vs. relax scripts across the three time points. Each dot is a participant, the horizontal line in the middle of the boxplot represents the median. On the right of each panel is the posterior distribution of the difference between the groups. The black line is the 95% HDI. **(b)** Average hippocampus reactivity to sad vs. relax in the ketamine group (blue) and the midazolam group (orange) in the three time points. Error bars represent standard error of the mean. Brain image depicts the mask of the hippocampus ROI (center: MNI [-28,-18,-16], [28,-18,-16]).

vmPFC:

Comparing the vmPFC activation while listening to the sad script (vs. neutral) revealed no groups differences after treatment [ketamine M=0.08 (sd=0.51], midaolzam: 0.09 (sd=0.51), mean posterior=-0.008 (sd=0.22) 95%HDI (-0.36,0.34)]; and in 30-day follow-up [ketamine M=-0.04 (sd=0.45], midaolzam: 0.04 (sd=0.31), mean posterior = -0.086 (sd=0.19) 95%HDI (-0.45,0.30)]. No difference was found between timepoints in the activation of vmPFC as well. see figure below.

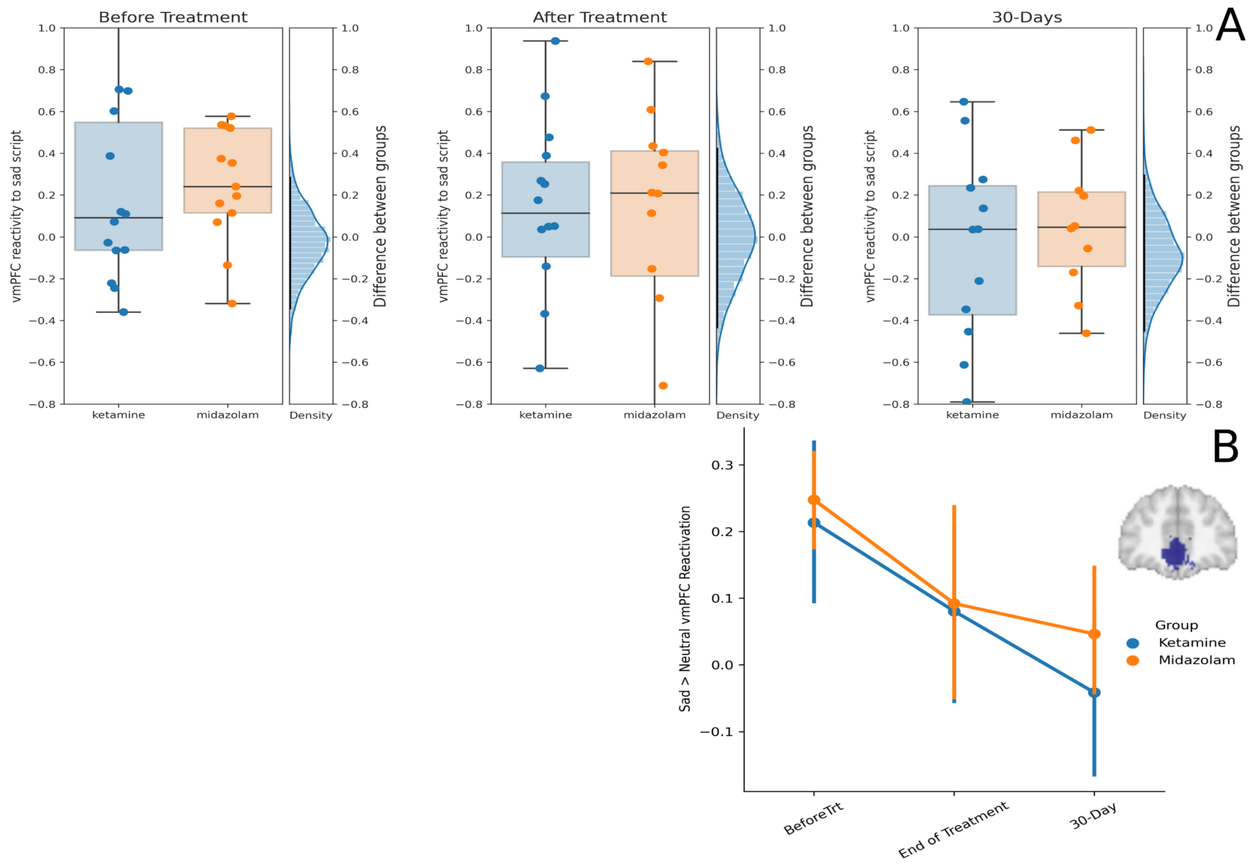

Figure S4: (a) Differences between the ketamine and midazolam groups in vmPFC reactivity to sad vs. relax scripts across the three time points. Each dot is a participant, the horizontal line in the middle of the boxplot represents the median. On the right of each panel is the posterior distribution of the difference between the groups. The black line is the 95% HDI. **(b)** Average vmPFC reactivity to sad vs. relax in the ketamine group (blue) and the midazolam group (orange) in the three time points. Error bars represent standard error of the mean. Brain image depicts the mask of the vmPFC ROI.

Results from main activation analysis:

vmPFC activation results:

Before treatment, average vmPFC reactivity to trauma vs neutral script was -0.09 (sd=0.75) in the ketamine group [N=14] and -0.03 (sd=0.54) in the midazolam group [N=13]. At the end of treatment (7 days after infusion) the average vmPFC reactivity was -0.03 (sd=0.37) in the ketamine group [N=14], and 0.07 (sd=0.46) in the midazolam group [N=12]. At 30-days follow-up the vmPFC reactivity was -0.01 (sd=0.48) in the ketamine group [N=12] and 0.01 (sd=0.34) in the midazolam group [N=10]. The statistical analysis showed no significant effect for time, also comparing between ketamine and midazolam groups (in each time point), revealed no difference. See Figure S1.

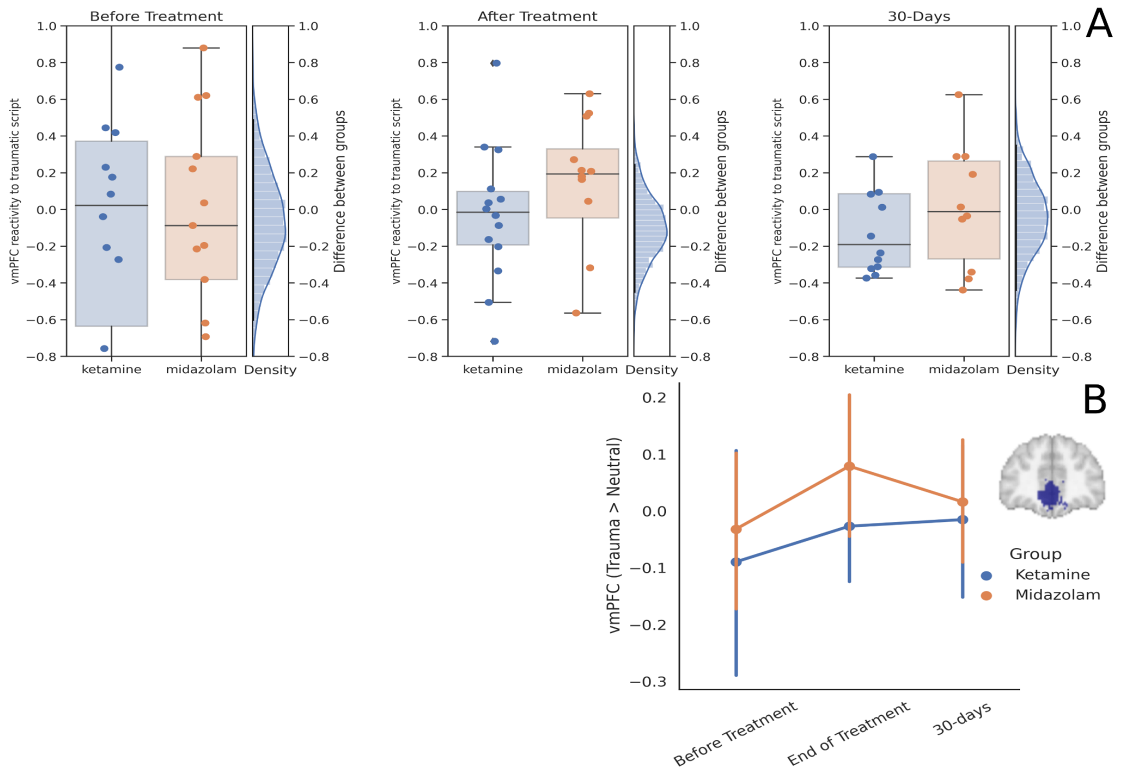

Figure S5: (a) Differences between the ketamine and midazolam groups in vmPFC reactivity to trauma vs. relax scripts across the three time points. Each dot is a participant, the horizontal line in the middle of the boxplot represents the median. On the right of each panel is the posterior distribution of the difference between the groups. The black line is the 95% HDI. **(b)** Average vmPFC reactivity to trauma vs. relax in the ketamine group (blue) and the midazolam group (orange) in the three time points. Error bars represent standard error of the mean. Brain image depicts the mask of the vmPFC ROI.

Changes in connectivity:

Amygdala-vmPFC:

amygdala-vmPFC functional connectivity was not significantly different between the groups after treatment [ketamine M=0.44 (sd=0.16)], midazolam: 0.47 (sd=0.16), mean posterior=-0.02 (sd=0.08) 95%HDI(-0.18,0.13)]; and in 30-day follow-up [ketamine M=0.37 (sd=0.24)], midazolam: 0.49 (sd=0.19), mean posterior=0.11 (sd=0.12) 95%HDI(-0.28,0.04)]. There was no time effect as well.

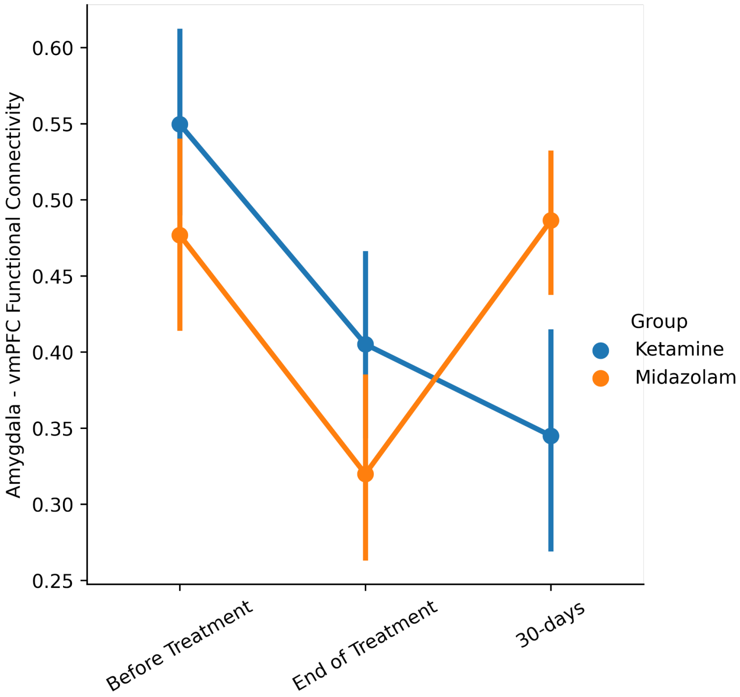

Figure S6: Functional connectivity between the amygdala and the vmPFC at each time point.

Amygdala-Anterior hippocampus connectivity:

Amygdala-anterior hippocampus functional connectivity was not significantly different between the groups after treatment [ketamine M=0.31 (sd=0.25], midazolam: 0.29 (sd=0.24), mean posterior=0.016 (sd=0.10) 95%HDI(-0.17,0.22)]; and in 30-day follow-up [ketamine M=0.32 (sd=0.24], midazolam: 0.20 (sd=0.29), mean posterior=0.12 (sd=0.13) 95%HDI(-0.13,0.36)]. There was no time effect as well.

Amygdala - posterior hippocampus:

Amygdala-posterior hippocampus functional connectivity was not significantly different between the groups after treatment [ketamine M=0.44 (sd=0.16], midazolam: 0.47 (sd=0.16), mean posterior=-0.05 (sd=0.11) 95%HDI(-0.27,0.17)]; and in 30-day follow-up [ketamine M=0.37 (sd=0.24], midazolam: 0.49 (sd=0.19), mean posterior=0.05 (sd=0.12) 95%HDI(-0.18,0.28)]. There was no time effect as well.

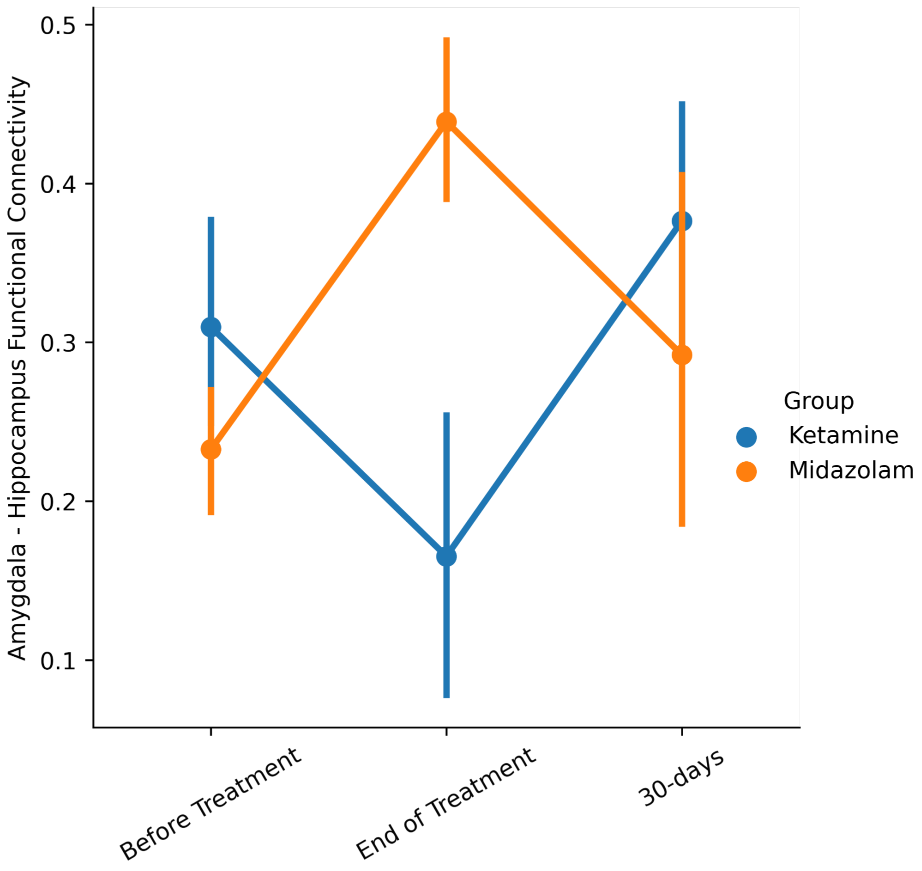

Figure S7: Functional connectivity between the amygdala and the posterior hippocampus at each time point.

Supplementary 3: Motion Parameters:

Table S1: Motion parameters

Variable Mean (∓SD)	Ketamine	Midazolam	Statistics (*df*),p
FD session 1	0.23 (∓0.1)	0.22 (∓0.10)	t(25) = 0.52, p = n.s.
FD session 2	0.26 (∓0.08)	0.23 (∓0.19)	t(23) = 0.72, p=n.s.
FD session 3	0.26 (∓0.15)	0.24 (∓0.14)	t(19) = 0.47, p=n.s.
DVARS session 1	59.72 (∓4.45)	55.9 (∓5.31)	t(25) = 1.88, p=0.07
DVARS session 2	61.07 (∓3.69)	57.93 (∓9.18)	t(23) = 1.60, p=n.s.
DVARS session 3	60.08 (∓6.01)	56.34 (∓8.81)	t(19) = 1.02, p=n.s.
Translation session 1	0.88 (∓ 0.34)	0.84 (∓0.22)	t(21) = 0.35, p=n.s.
Translation session 2	0.86 (∓ 0.28)	1.03 (∓0.25)	t(21) = 1.48, p=n.s.
Translation session 3	0.98 (∓ 0.35)	0.81 (∓0.24)	t(19) = 1.36, p=n.s.
Rotation session 1	0.007 (∓ 0.002)	0.006 (∓0.002)	t(21) = 0.48, p=n.s.
Rotation session 2	0.007 (∓ 0.002)	0.008 (∓0.002)	t(21) = 1.01, p=n.s.
Rotation session 3	0.007 (∓ 0.003)	0.006 (∓0.002)	t(19) = 1.3, p=n.s.

Supplement 4: Power Analysis:

To test the ability of our current sample size to distinguish the difference between the groups (ketamine/midazolam) in activation of our regions of interest (i.e., amygdala, hippocampus, and vmPFC), we have simulated data in three sizes: N=20, N=25, and N=30. Groups were divided into two, with ketamine at 52% and midazolam at 48% (as groups are not equal in our original data as well). We have generated data using three steps: first, randomly sample from a normal distribution (with mean=0 and standard deviation of 0.3; similar to our data) to both groups (to receive baseline activation). We then generated a delta between the baseline and end of treatment. This was generated using two different distributions, one for the ketamine (mean=-.2, sd=.3), and one for the midazolam (mean=0, sd=.3). This assumes a decline in the ketamine group, similar to what we expect, and no change in the midazolam group. We then calculated the activation of each simulated participant's ROI in the second observation (i.e., end of treatment) by adding the delta to the ROI at baseline. We used the same procedure for the second delta (30 days follow-up), but this time, both groups' delta was randomly sampled from a normal distribution with mean=0 and sd=.3). Next, we ran our statistical model (see main) 1000 iterations for each sample size. We calculated the number of times in which the posterior distribution's 95% interval at time 2 (i.e., end of treatment) crossed zero. When N=20, 12.7% of the times, the posterior distribution crossed zero. When N=25 it was 11.0%, and in N=30 it was 9.3%. This suggests a (one-tailed) power above 90% for our sample size.

Supplement 5: Preprocessing

Results included in this manuscript come from preprocessing performed using *fMRIPrep* 1.5.8 (Esteban, Markiewicz, et al. (2018); Esteban, Blair, et al. (2018); RRID:SCR_016216), which is based on *Nipype* 1.4.1 (Gorgolewski et al. (2011); Gorgolewski et al. (2018); RRID:SCR_002502).

Anatomical data preprocessing

Each participant had a total of 4 T1-weighted (T1w) images (one for each session). All of them were corrected for intensity non-uniformity (INU) with N4BiasFieldCorrection (Tustison et al. 2010), distributed with ANTs 2.2.0 (Avants et al. 2008, RRID:SCR_004757). The T1w-reference was then skull-stripped with a *Nipype* implementation of the antsBrainExtraction.sh workflow (from ANTs), using OASIS30ANTs as target template. Brain tissue segmentation of cerebrospinal fluid (CSF), white-matter (WM) and gray-matter (GM) was performed on the brain-extracted T1w using fast (FSL 5.0.9, RRID:SCR_002823, Zhang, Brady, and Smith 2001). A T1w-reference map was computed after registration of 4 T1w images (after INU-correction) using mri_robust_template (FreeSurfer 6.0.1, Reuter, Rosas, and Fischl 2010). Brain surfaces were reconstructed using recon-all (FreeSurfer 6.0.1, RRID:SCR_001847, Dale, Fischl, and Sereno 1999), and the brain mask estimated previously was refined with a custom variation of the method to reconcile ANTs-derived and FreeSurfer-derived segmentations of the cortical gray-matter of Mindboggle (RRID:SCR_002438, Klein et al. 2017). Volume-based spatial normalization to two standard spaces (MNI152NLin2009cAsym, MNI152NLin6Asym) was performed through nonlinear registration with antsRegistration (ANTs 2.2.0), using brain-extracted versions of both T1w reference and the T1w template. The following templates were selected for spatial normalization: *ICBM 152 Nonlinear Asymmetrical template version 2009c* [Fonov et al. (2009), RRID:SCR_008796; TemplateFlow ID: MNI152NLin2009cAsym], *FSL's MNI ICBM 152 non-linear 6th Generation Asymmetric Average Brain Stereotaxic Registration Model* [Evans et al. (2012), RRID:SCR_002823; TemplateFlow ID: MNI152NLin6Asym].

Functional data preprocessing

For each of the BOLD runs found per subject (across all tasks and sessions), the following preprocessing was performed. First, a reference volume and its skull-stripped version were generated using a custom methodology of *fMRIPrep*. Susceptibility distortion correction (SDC) was omitted. The BOLD reference was then co-registered to the T1w reference using bbregister (FreeSurfer) which implements boundary-based registration (Greve and Fischl 2009). Co-registration was configured with six degrees of freedom. Head-motion parameters with respect to the BOLD reference (transformation matrices, and six corresponding rotation and translation parameters) are estimated before any spatiotemporal filtering using mcflirt (FSL 5.0.9, Jenkinson et al. 2002). BOLD runs were slice-time corrected using 3dTshift from AFNI 20160207 (Cox and Hyde 1997, RRID:SCR_005927). The BOLD time-series, were resampled to surfaces on the following spaces: *fsnative*, *fsaverage5*. *Grayordinates* files (Glasser et al. 2013), which combine surface-sampled data and volume-sampled data, were also generated. The BOLD time-series (including slice-timing correction when applied) were resampled onto their original, native space by applying the transforms to correct for head-motion. These resampled BOLD time-series will

be referred to as *preprocessed BOLD in original space*, or just *preprocessed BOLD*. The BOLD time-series were resampled into several standard spaces, correspondingly generating the following *spatially-normalized, preprocessed BOLD runs*: MNI152NLin2009cAsym, MNI152NLin6Asym. First, a reference volume and its skull-stripped version were generated using a custom methodology of *fMRIPrep*. Automatic removal of motion artifacts using independent component analysis (ICA-AROMA, Pruim et al. 2015) was performed on the *preprocessed BOLD on MNI space* time-series after removal of non-steady state volumes and spatial smoothing with an isotropic, Gaussian kernel of 6mm FWHM (full-width half-maximum). Corresponding "non-aggresively" denoised runs were produced after such smoothing. Additionally, the "aggressive" noise-regressors were collected and placed in the corresponding confounds file. Several confounding time-series were calculated based on the *preprocessed BOLD*: framewise displacement (FD), DVARS and three region-wise global signals. FD and DVARS are calculated for each functional run, both using their implementations in *Nipype* (following the definitions by Power et al. 2014). The three global signals are extracted within the CSF, the WM, and the whole-brain masks. Additionally, a set of physiological regressors were extracted to allow for component-based noise correction (*CompCor*, Behzadi et al. 2007). Principal components are estimated after high-pass filtering the *preprocessed BOLD* time-series (using a discrete cosine filter with 128s cut-off) for the two *CompCor* variants: temporal (tCompCor) and anatomical (aCompCor). tCompCor components are then calculated from the top 5% variable voxels within a mask covering the subcortical regions. This subcortical mask is obtained by heavily eroding the brain mask, which ensures it does not include cortical GM regions. For aCompCor, components are calculated within the intersection of the aforementioned mask and the union of CSF and WM masks calculated in T1w space, after their projection to the native space of each functional run (using the inverse BOLD-to-T1w transformation). Components are also calculated separately within the WM and CSF masks. For each CompCor decomposition, the *k* components with the largest singular values are retained, such that the retained components' time series are sufficient to explain 50 percent of variance across the nuisance mask (CSF, WM, combined, or temporal). The remaining components are dropped from consideration. The head-motion estimates calculated in the correction step were also placed within the corresponding confounds file. The confound time series derived from head motion estimates and global signals were expanded with the inclusion of temporal derivatives and quadratic terms for each (Satterthwaite et al. 2013). Frames that exceeded a threshold of 0.5 mm FD or 1.5 standardised DVARS were annotated as motion outliers. All resamplings can be performed with *a single interpolation step* by composing all the pertinent transformations (i.e. head-motion transform matrices, susceptibility distortion correction when available, and co-registrations to anatomical and output spaces). Gridded (volumetric) resamplings were performed using antsApplyTransforms (ANTs), configured with Lanczos interpolation to minimize the smoothing effects of other kernels (Lanczos 1964). Non-gridded (surface) resamplings were performed using mri_vol2surf (FreeSurfer).

Many internal operations of *fMRIPrep* use *Nilearn* 0.6.1 (Abraham et al. 2014, RRID:SCR_001362), mostly within the functional processing workflow. For more details of the pipeline, see the section corresponding to workflows in *fMRIPrep*'s documentation.

Copyright Waiver
The above boilerplate text was automatically generated by fMRIPrep with the express intention that users should copy and paste this text into their manuscripts *unchanged*. It is released under the CC0 license.

References:

Abraham, Alexandre, Fabian Pedregosa, Michael Eickenberg, Philippe Gervais, Andreas Mueller, Jean Kossaifi, Alexandre Gramfort, Bertrand Thirion, and Gael Varoquaux. 2014. “Machine Learning for Neuroimaging with Scikit-Learn.” *Frontiers in Neuroinformatics* 8. https://doi.org/10.3389/fninf.2014.00014.

Avants, B.B., C.L. Epstein, M. Grossman, and J.C. Gee. 2008. “Symmetric Diffeomorphic Image Registration with Cross-Correlation: Evaluating Automated Labeling of Elderly and Neurodegenerative Brain.” *Medical Image Analysis* 12 (1): 26–41. https://doi.org/10.1016/j.media.2007.06.004.

Behzadi, Yashar, Khaled Restom, Joy Liau, and Thomas T. Liu. 2007. “A Component Based Noise Correction Method (CompCor) for BOLD and Perfusion Based fMRI.” *NeuroImage* 37 (1): 90–101. https://doi.org/10.1016/j.neuroimage.2007.04.042.

Cox, Robert W., and James S. Hyde. 1997. “Software Tools for Analysis and Visualization of fMRI Data.” *NMR in Biomedicine* 10 (4-5): 171–78. https://doi.org/10.1002/(SICI)1099-1492(199706/08)10:4/5<171::AID-NBM453>3.0.CO;2-L.

Dale, Anders M., Bruce Fischl, and Martin I. Sereno. 1999. “Cortical Surface-Based Analysis: I. Segmentation and Surface Reconstruction.” *NeuroImage* 9 (2): 179–94. https://doi.org/10.1006/nimg.1998.0395.

Esteban, Oscar, Ross Blair, Christopher J. Markiewicz, Shoshana L. Berleant, Craig Moodie, Feilong Ma, Ayse Ilkay Isik, et al. 2018. “FMRIPrep.” *Software*. Zenodo. https://doi.org/10.5281/zenodo.852659.

Esteban, Oscar, Christopher Markiewicz, Ross W Blair, Craig Moodie, Ayse Ilkay Isik, Asier Erramuzpe Aliaga, James Kent, et al. 2018. “fMRIPrep: A Robust Preprocessing Pipeline for Functional MRI.” *Nature Methods*. https://doi.org/10.1038/s41592-018-0235-4.

Evans, AC, AL Janke, DL Collins, and S Baillet. 2012. “Brain Templates and Atlases.” *NeuroImage* 62 (2): 911–22. https://doi.org/10.1016/j.neuroimage.2012.01.024.

Fonov, VS, AC Evans, RC McKinstry, CR Almli, and DL Collins. 2009. “Unbiased Nonlinear Average Age-Appropriate Brain Templates from Birth to Adulthood.” *NeuroImage* 47, Supplement 1: S102. https://doi.org/10.1016/S1053-8119(09)70884-5.

Glasser, Matthew F., Stamatios N. Sotiropoulos, J. Anthony Wilson, Timothy S. Coalson, Bruce Fischl, Jesper L. Andersson, Junqian Xu, et al. 2013. “The Minimal Preprocessing Pipelines for the Human Connectome Project.” *NeuroImage*, Mapping the connectome, 80: 105–24. https://doi.org/10.1016/j.neuroimage.2013.04.127.

Gorgolewski, K., C. D. Burns, C. Madison, D. Clark, Y. O. Halchenko, M. L. Waskom, and S. Ghosh. 2011. “Nipype: A Flexible, Lightweight and Extensible Neuroimaging Data Processing Framework in Python.” *Frontiers in Neuroinformatics* 5: 13. https://doi.org/10.3389/fninf.2011.00013.

Gorgolewski, Krzysztof J., Oscar Esteban, Christopher J. Markiewicz, Erik Ziegler, David Gage

Ellis, Michael Philipp Notter, Dorota Jarecka, et al. 2018. “Nipype.” *Software*. Zenodo. https://doi.org/10.5281/zenodo.596855.
Greve, Douglas N, and Bruce Fischl. 2009. “Accurate and Robust Brain Image Alignment Using Boundary-Based Registration.” *NeuroImage* 48 (1): 63–72. https://doi.org/10.1016/j.neuroimage.2009.06.060.
Jenkinson, Mark, Peter Bannister, Michael Brady, and Stephen Smith. 2002. “Improved Optimization for the Robust and Accurate Linear Registration and Motion Correction of Brain Images.” *NeuroImage* 17 (2): 825–41. https://doi.org/10.1006/nimg.2002.1132.
Klein, Arno, Satrajit S. Ghosh, Forrest S. Bao, Joachim Giard, Yrjö Häme, Eliezer Stavsky, Noah Lee, et al. 2017. “Mindboggling Morphometry of Human Brains.” *PLOS Computational Biology* 13 (2): e1005350. https://doi.org/10.1371/journal.pcbi.1005350.
Lanczos, C. 1964. “Evaluation of Noisy Data.” *Journal of the Society for Industrial and Applied Mathematics Series B Numerical Analysis* 1 (1): 76–85. https://doi.org/10.1137/0701007.
Power, Jonathan D., Anish Mitra, Timothy O. Laumann, Abraham Z. Snyder, Bradley L. Schlaggar, and Steven E. Petersen. 2014. “Methods to Detect, Characterize, and Remove Motion Artifact in Resting State fMRI.” *NeuroImage* 84 (Supplement C): 320–41. https://doi.org/10.1016/j.neuroimage.2013.08.048.
Pruim, Raimon H. R., Maarten Mennes, Daan van Rooij, Alberto Llera, Jan K. Buitelaar, and Christian F. Beckmann. 2015. “ICA-AROMA: A Robust ICA-Based Strategy for Removing Motion Artifacts from fMRI Data.” *NeuroImage* 112 (Supplement C): 267–77. https://doi.org/10.1016/j.neuroimage.2015.02.064.
Reuter, Martin, Herminia Diana Rosas, and Bruce Fischl. 2010. “Highly Accurate Inverse Consistent Registration: A Robust Approach.” *NeuroImage* 53 (4): 1181–96. https://doi.org/10.1016/j.neuroimage.2010.07.020.
Satterthwaite, Theodore D., Mark A. Elliott, Raphael T. Gerraty, Kosha Ruparel, James Loughead, Monica E. Calkins, Simon B. Eickhoff, et al. 2013. “An improved framework for confound regression and filtering for control of motion artifact in the preprocessing of resting-state functional connectivity data.” *NeuroImage* 64 (1): 240–56. https://doi.org/10.1016/j.neuroimage.2012.08.052.
Tustison, N. J., B. B. Avants, P. A. Cook, Y. Zheng, A. Egan, P. A. Yushkevich, and J. C. Gee. 2010. “N4ITK: Improved N3 Bias Correction.” *IEEE Transactions on Medical Imaging* 29 (6): 1310–20. https://doi.org/10.1109/TMI.2010.2046908.
Zhang, Y., M. Brady, and S. Smith. 2001. “Segmentation of Brain MR Images Through a Hidden Markov Random Field Model and the Expectation-Maximization Algorithm.” *IEEE Transactions on Medical Imaging* 20 (1): 45–57. https://doi.org/10.1109/42.906424.

Combining neurobiology and new learning: Ketamine and Prolonged exposure: A potential rapid treatment for PTSD

<u>Preliminary Clinical Study – Proof of principal study in a small number of subjects to see if efficacy is detected with the new treatment:</u> Posttraumatic Stress Disorder (PTSD) is a debilitating and at chronic mental illness. There are currently only two FDA-approved medications for the treatment of PTSD, both of which may take weeks to months to reach full clinical effects. The rates of nonresponse to these selective serotonin reuptake inhibitor antidepressants are high. Therefore, there is a tremendous need to test novel pharmacological approaches to PTSD. Trauma focus psychotherapies on the other hand require a high level of commitment by attending weekly visits for a substantial amount of time. An average course of Prolonged Exposures therapy (PE) or Trauma focus CBT usually lasts about 3 months until completion with about 35% of completers being classified as non-responders. Moreover, naturalistic studies suggest that more than 60% drop out of therapy before receiving appropriate dose to experience a significant symptoms reduction (Harpaz-Rotem & Rosenheck, 2011).

Recently it was reported in an RCT that 7-day intensive trauma focus psychotherapy was equally effective as a full course of 3-month weekly dose of the same therapy (Ehlers, A. et al., 2014). There are also accumulating evidences to indicate that the N-methyl-D-aspartate (NMDA) glutamate receptor antagonist, ketamine, produces a rapid antidepressant action within 4 hours of administration, with a short-term response rate ranging from 43% to 90% (Murrough, J. et al., 2013). This antidepressant effect was sustained for up to 7-28 days after a single ketamine infusion. Evidence also indicates that a ketamine-induced surge in glutamate neurotransmission, particularly in the frontal cortex, plays a crucial role in the antidepressant and psychotomimetic effects of the drug. To date, more than 15 studies have investigated the efficacy of 0.5mg/kg ketamine infusion in more than 300 depressed patients. While these data strongly support the efficacy of ketamine in depression, the impact of ketamine in the treatment of PTSD was not yet well investigated. To date, only one RCT was conducted to show positive effect of ketamine insignificantly reducing PTSD symptoms compared to placebo (Feder, A. et al., 2014).

Recent animal studies have begun to elucidate downstream effects of ketamine that may underlie its beneficial effects in both depressed and PTSD patients. Uncontrollable stress in animals produces a syndrome that, at both biological and behavioral levels, resembles PTSD. These stressors initially activate glutamate circuits and trigger pro-inflammatory processes that initiate a cascade of neural events that impair functional and structural glutamatergic connectivity, contributing to the long-term effects of stress (Duman, R. and Aghajanian, G., 2012; Sanacora et al., 2008). The changes include: 1) glial loss with resulting elevations in extra-synaptic glutamate levels, 2) suppression of glutamate neural activity due to overstimulation of presynaptic metabotropic glutamate receptor-2 (mGluR2) receptors, and 3) overstimulation of GluN2B (NR2B) subunit-containing extra-synaptic NMDA receptors. These effects combine to cause loss of dendritic spines and dendritic retraction in cortico-limbic circuits regulating mood. Recent animal studies have also begun to elucidate downstream effects of ketamine that may underlie its beneficial effects in depressed patients (Duman and Aghajanian, 2012). NMDA receptor antagonist effects drive a rapid and profound growth of functional dendritic spines that enhance neuroplasticity and restore functional neural connectivity disrupted by stress (Duman and Aghajanian, 2012). In sum, convergent evidence suggests that, at subanesthetic doses, ketamine's antagonism of NMDA receptors rapidly triggers 3 consecutive events: 1) a presynaptic disinhibition of glutamatergic neurons leading to a synaptic glutamate surge, 2) an increased activation of AMPA receptors, and 3) a post-synaptic activation of neuroplasticity-related signaling pathways resulting in overall synaptogenesis and synaptic potentiation.

Based on the current research findings on the therapeutic effectiveness of trauma focus psychotherapy and of ketamine, combining the two treatments may yield a promising new rapid 7-day treatment for PTSD. As PTSD symptoms' structure is comprised of several unique clusters which include re-experiencing, avoidance, numbing/depression and hypervigilance we hypothesize that by combining Ketamine with PE we can address these symptoms clusters more effectively. This treatment has the potential to produce a significant therapeutic effect that otherwise would take months to occur by tapping on the enhanced neuroplasticity and the antidepressant effect of ketamine (which lasts between 24hrs to 7 days), to promote rapid changes in learning and memory using prolonged exposure therapy within this unique "window of opportunity".

More specifically, PE has two major components of therapeutic action – imaginal exposure to process the traumatic memories and in-vivo exposure exercises to reduce avoidance and exaggerated startle response by

confronting them within a safe environment. Thus, we propose to employ prolonged exposure therapy (PE), the most effective and well documented treatment for PTSD, during the period of enhanced neuroplasticity following a single infusion of Ketamine. Imaginal exposure will enhance memory reconsolidation of the traumatic memories, leading to reduction of re-experiencing symptoms, while in vivo exposure will reduce avoidant behavior and exaggerated startle responses.

We will also use 3T MRI scanner to assess biomarkers of structural connectivity (using DTI) and fMRI for biomarkers of functional connectivity and hyper-reactivity to trauma reminders. DTI has been used to study PTSD, where connectivity deficits have been described in cortico-limbic structures that do not appear to be dependent upon a diagnosis of traumatic brain injury (Admon et al., 2013). PTSD is also associated with differences in activation or in functional connectivity in the amygdala, striatum, insula, anterior cingulate cortex, hippocampus and prefrontal cortex (Shin, L. & Liberzon, I., 2010). It was found that ketamine enhances functional connectivity at rest, but reduces working memory task-related activation and connectivity and the functional antagonism between the executive and default mode networks (Driesen N. et al., 2013). We propose to evaluate functional connectivity using f-MRI in the current study to test the following hypotheses: 1) Alterations in functional connectivity (hyperconnectivity of amygdala-insula-cingulate and reduced DLPFC-amygdala connectivity prior to treatment, will predict treatment response; and 2) Ketamine will normalize these changes to better understand this type of functional relationship between the PFC and amygdala. It is also important to link the connectivity changes in humans to alterations in brain activation during trauma cue exposure. Thus, we will also use personal trauma narratives during fMRI scans (from the PE) to assess level of brain activation during trauma reminders. Prior studies in PTSD have suggested that patterns of cortico-limbic functional connectivity are associated with circuit reactivity to traumatic reminders and anticipation of negative stimuli (Fonzo et al., 2010).

Using a biomarker-informed, double blind placebo-controlled design, the present proposal aims to examine the efficacy of a single dose of ketamine infusion, as compared to midazolam, that will be combined with an intensive one week PE, in producing a rapid and sustained reduction in PTSD symptomatology in veterans. In addition, we propose use of state-of-the-art neuroimaging assessments at baseline and at the end of treatment trial to gain insight into the neurobiology of PTSD and the neural mechanisms dictating treatment response or resistance. **Aim 1:** To demonstrate the beneficial effects of combining low dose ketamine (0.5 mg/kg infused over 40 min) and PE on PTSD symptoms as assessed by structured interviews and behavioral ratings in the OIF/OEF/OND population. A mixed effects regression model will reveal a significant group by time interaction, with rapid reduction in PTSD symptoms in the ketamine group compared to midazolam and placebo groups. **Aim 2:** To assess the functional connectivity and hyper-reactivity in the "mood" and "memory" network which include: the amygdala, striatum, insula, anterior cingulate cortex, hippocampus and prefrontal cortex in pre and post treatment task listening to the trauma narrative/neutral narrative/and a significant non-traumatic memory narrative pre and post treatment. **Aim 3:** To demonstrate the ability of ketamine treatment to restore structural connectivity by using diffusion weighted imaging (DTI) and global probabilistic tractography with anatomical priors, we will estimate the cingulum fractional anisotropy (FA) at baseline, and post treatment.

Procedure: We will target OIF/OEF/OND veterans entering new episode of PTSD treatment who are medication free for at least 60 days and are not diagnosed with substance use disorder, schizophrenia and/or bipolar disorder. Subjects will be screened for PTSD using the CAPS and the SCID for other comorbid condition. All veterans who will meet the diagnostic criteria for PTSD and will demonstrated sever PTSD as measured by CAPS score above 50 will be randomized to either the 7-day PE+ ketamine group or the control group which will receive 7-day PE+ midazolam. After session 1 of the PE when the most significant trauma was identified trauma script will be prepared for the MRI procedure. Following session 2 (which is educational), baseline MRI and fMRI will be conducted. At PE session 3, when the traumatic memory is first processed within a safe context – the traumatic memory is brought into a labile state. Thus, at the end of session 3, ketamine or midazolam will be infused to allow alternations the reconsolidation process to occur more rapidly tapping on the ketamine's neuroplasticity effect. As ketamine therapeutic effect lasts up to a week, 24 hours after the infusion PE sessions 4-7 will continue daily to exercise in-vivo exposure to reduce avoidance and hypervigilance and imaginal exposure processing the traumatic memory will continue to ensure the modified emotional reconsolidation of the traumatic memory. Post treatment MRI and fMRI will follow PE session seven. 30 days follow-up and 120 days follow-up will be conducted to measure PTSD symptoms' severity and the long-term effects of treatment.

References:

1. Harpaz-Rotem, I. & Rosenheck, R. (2011). Serving the one who served: Retention of Newly Returning Veterans from Iraq and Afghanistan in Mental Health Treatment. *Psychiatric Services, 62,* 22-27
2. Ehlers, A. et al., (2014). A randomized controlled trial of 7-day intensive and standard weekly cognitive therapy for PTSD and emotion-focused supportive therapy. *American Journal of Psychiatry, 171,* 294-304
3. Murrough, J. et al. (2013). Antidepressant efficacy of ketamine in treatment-resistant major depression: a two-site randomized controlled trial. *American Journal of Psychiatry, 170,* 1134-1142
4. Feder, A. et al. (2014). Efficacy of intravenous ketamine for treatment of chronic posttraumatic stress disorder: a randomized clinical trial. *JAMA Psychiatry, 71,* 681-8
5. Duman, R. and Aghajanian, G. (2012). Synaptic dysfunction in depression: potential therapeutic targets. *Science, 338,* 68-72
6. Sanacora, G. et al. (2008). Targeting the glutamatergic system to develop novel, improved therapeutics for mood disorders. *Nature Reviews: Drug Discovery 7,* 426-37
7. Admon, R. et al. (2013). Stress-induced reduction in hippocampal volume and connectivity with the ventromedial prefrontal cortex are related to maladaptive responses to stressful military service. *Hum Brain Mapp, 34,* 2808-16
8. Shin, L. & Liberzon, I. (2010). The neurocircuitry of fear, stress, and anxiety disorders. Neuropsychopharmacology, 35, 169-91
9. Driesen N. et al. (2013). Relationship of resting brain hyperconnectivity and schizophrenia-like symptoms produced by the NMDA receptor antagonist ketamine in humans. *Mol Psychiatry, 18,* 1199-204
10. Fonzo, G.A. et al. (2010). Exaggerated and disconnected insular-amygdalar blood oxygenation level-dependent response to threat-related emotional faces in women with intimate-partner violence posttraumatic stress disorder. Biological psychiatry, 68, 433-41
